# Bioactive Lipids in *Dunaliella salina*: Implications for Functional Foods and Health

**DOI:** 10.3390/foods13203321

**Published:** 2024-10-19

**Authors:** Rita Pais, Tiago Conde, Bruna B. Neves, Marisa Pinho, Marta Coelho, Hugo Pereira, Alexandre M. C. Rodrigues, Pedro Domingues, Ana Maria Gomes, Ralph Urbatzka, Rosário Domingues, Tânia Melo

**Affiliations:** 1 CESAM—Centre for Environmental and Marine Studies, Department of Chemistry, University of Aveiro, Santiago University Campus, 3810-193 Aveiro, Portugal; ana.s.pais@ua.pt (R.P.); tiagoalexandreconde@ua.pt (T.C.); brunabneves@ua.pt (B.B.N.); marisapinho@ua.pt (M.P.); mrd@ua.pt (R.D.); 2 Mass Spectrometry Centre, LAQV-REQUIMTE, Department of Chemistry, University of Aveiro, Santiago University Campus, 3810-193 Aveiro, Portugal; p.domingues@ua.pt; 3 CBQF—Centro de Biotecnologia e Química Fina, Laboratório Associado, Escola Superior de Biotecnologia, Universidade Católica Portuguesa, Rua Diogo Botelho 1327, 4169-005 Porto, Portugal; mcoelho@ucp.pt (M.C.); amgomes@ucp.pt (A.M.G.); 4 GreenCoLab—Associação Oceano Verde, University of Algarve, Campus de Gambelas, 8005-139 Faro, Portugal; hugopereira@greencolab.com; 5 Necton, S.A., Belamandil, 8700-152 Olhão, Portugal; alexandre.rodrigues@necton.pt; 6 Biodiscovery for Health Group, CIIMAR/CIMAR, Interdisciplinary Centre of Marine and Environmental Research, Terminal de Cruzeiros do Porto de Leixões, University of Porto, 4450-208 Matosinhos, Portugal; rurbatzka@ciimar.up.pt

**Keywords:** microalgae, food, lipidomics, lipids, fatty acids

## Abstract

*Dunaliella salina* is a green microalga extensively explored for β-carotene production, while knowledge of its lipid composition is still limited and poorly investigated. Among lipids, polar lipids have been highlighted as bioactive phytochemicals with health-promoting properties. This research aimed to provide an in-depth lipidome profiling of *D. salina* using liquid and gas chromatography coupled with mass spectrometry. The lipid content was 6.8%, including phospholipids, glycolipids, betaine lipids, sphingolipids, triglycerides, diglycerides, and pigments. Among the total esterified fatty acids, 13.6% were 18:3 omega-3 and 14.7% were 18:1 omega-9. The lipid extract of *D. salina* showed anti-inflammatory activity by inhibiting cyclooxygenase-2 activity at 100 µg/mL, dose-dependent antioxidant scavenging activity, and antidiabetic activity by inhibiting α-glucosidase activity at 25 and 125 µg/mL. In conclusion, the lipid extract of *D. salina* has the potential to be used as a functional food ingredient or in the nutraceutical and cosmeceutical industries.

## 1. Introduction

Unhealthy diets and poor eating habits, either due to an excess of food or a shortage of essential nutrients, are major causes of non-communicable diseases (NCD), a burden in Western society. A high intake of saturated fats and trans fatty acids (FA), as well as a deficit in omega-3 polyunsaturated fatty acids (PUFA), is among the nutrient imbalances that enhance age-related disorders and NCDs, namely cardiovascular diseases and diabetes [1].

Microalgae emerged as alternative sources of lipids, especially omega-3 lipids that are typically supplied from fish or fish oil, but overfishing is a major drawback for these resources. Additionally, it addresses the growing trend of people following vegan/vegetarian diets, requiring plant-based sources of essential lipids, and demand for alternative and sustainable solutions to provide these healthy lipids. Algae are rich in essential nutrients and bioactive compounds, including lipids, and are recommended for sustainable and healthy diets, in line with some initiatives launched by the European Union [2]. Although lipids from algae have been reported with bioactive properties, these remain mostly associated with FAs, which are mainly esterified to complex lipids, such as triglycerides (TG), as well as to membrane phospholipids (PLs) and chloroplast glycolipids (GLs). Algae PLs and GLs are being increasingly highlighted as the most active biomolecules with functional properties such as anti-inflammatory [3], anti-obesity [4], and antidiabetic activities [5], contributing to algal added-value and novel biotechnological applications.

Green microalgae, including the genus *Dunaliella*, offer fascinating features that can be used for biotechnology and industry-oriented applications, and thus there is an increasing interest in these microalgae. *Dunaliella* species are very resilient, with high adaptability to both high salinity and high light intensities, are produced industrially, and can produce several metabolites of interest [6]. Particularly, *Dunaliella salina* is a well-known reservoir of high-value biomolecules such as carotenoids, lipids, and FA 18:1 *n*-9 (oleic acid, OA), FA 18:3 *n*-3 (α-linolenic acid, ALA), and FA 18:2 *n*-6 (linoleic acid, LA) [7,8]. Among the various applications, and although it is already approved as a food supplement, this microalga is mostly explored for β-carotene production [9]. Interestingly, in *D. salina*, the lipid content can range from 7–60%, depending on the cultivation method and harvesting phase [10], thus evidencing that it can be explored as a source of lipids. However, its lipidome has not yet been characterized, so its valorization as a source of functional lipids has been neglected.

The interest in profiling algae lipidomes to unravel their added value and to promote their use as food and food ingredients for nutraceuticals is increasing. The lipidomes of some algae have been successfully identified by advanced lipidomics approaches to unravel the relationship between composition and biological value. In addition, omega-3 PUFAs bound to polar lipids, namely PL and GL, have intrinsic bioactive properties as well as increased bioavailability, making them more bio-effective [11]. Thus, microalgae polar lipids are of utmost importance, although current knowledge is still limited due to their chemical diversity and the lack of knowledge on microalgae lipidomes. Hence, the aim of this study was to characterize the lipidome of *D. salina* and bioprospect its lipids for potential value addition to its biomass.

Herein, detailed lipid and FA profiling of *D. salina* was determined using advanced lipidomics approaches (GC-MS and LC-MS), and its added value as a source of functional and valuable lipids with bioactive properties such as anti-inflammatory, antioxidant, and antidiabetic activities was evaluated using in chemico assays.

## 2. Materials and Methods

### 2.1. Reagents

High-performance liquid chromatography (HPLC)-grade dichloromethane, methanol, and ethanol were purchased from Fisher Scientific Ltd. (Loughborough, UK). Milli-Q purified water was used (Synergy^®^, Millipore Corporation, Billerica, MA, USA). The α,α-diphenyl-β-picryl-hydrazyl radicals (DPPH^•^) were purchased from Aldrich (Milwaukee, WI, USA) and the 2,2-azino-bis (3-ethylbenzothiazoline-6-sulfonic acid) radical cations (ABTS^•+^) from Fluka (Buchs, Switzerland). The remaining reagents were obtained from primary commercial sources. All lipid internal standards were purchased from Avanti Polar Lipids, Inc. (Alabaster, AL, USA).

### 2.2. Microalgae Material

The microalgae biomass was provided by Necton S.A. (Olhão, Portugal). Microalgae were grown in closed flat panel photobioreactors and kept monoalgal by mechanical and chemical pre-treatments of the water used for algal production, namely through ultrafiltration at 0.02 µm, sodium hypochlorite treatment at 200 ppm, and UV treatment. The culture was harvested by centrifugation, packed, and frozen at −20 °C. Before the experiments, samples were freeze-dried, reduced to powder, and stored in the dark at −20 °C.

### 2.3. Total Lipid Extraction

Lipids were extracted using a modified Folch’s method, as previously described [12]. Briefly, 25 mg of biomass was extracted with 2 mL of dichloromethane and methanol (2:1, *v*/*v*), vortexed for 2 min, centrifuged (PRO-Analytical, Centurion Scientific, Chichester, UK) at 2000 rpm for 10 min, and the supernatant was collected in a new tube. This process was repeated four times. The combined supernatants were dried under a nitrogen stream. A volume of 2 mL dichloromethane, 1 mL methanol, and 0.75 mL of Milli-Q was added, and samples were vortexed for 2 min and centrifuged at 2000 rpm for 10 min. The organic phase was collected in a new tube while the aqueous phase was re-extracted twice using 2 mL dichloromethane. The combined organic phases were dried under nitrogen steam. The lipid extracts were transferred to pre-weighed amber vials using 1.2 mL of dichloromethane. The amber vials were previously oven-dried (Binder, Tuttlingen, Germany) at 100 °C for two hours and cooled for thirty minutes on a desiccator, prior to being weighed. The total lipid content was estimated as a percentage of dry weight (DW) and the results were expressed as mean ± standard deviation of 5 replicates.

(1) $$Lipid\;yield\left(\%\;DW,\frac{w}{w}\right)=\frac{weight\;of\;lipid\;extract\;(mg)}{weight\;of\;biomass\;(mg)}\times 100,$$

### 2.4. Quantification of Phospholipids

Phospholipids present in the lipid extract were quantified according to a modified version of the Bartlett and Lewis method, as described before [13]. Briefly, the phosphorus present in 25 µg of lipid extract, obtained after the hydrolysis of PL with perchloric acid (70%) at 180 °C for 1 h, formed a complex with molybdate (2.5%), which further reacted with ascorbic acid (10%) at 100 °C for 5 min, and the absorbance of the new colored complex formed was measured at 797 nm using a UV/vis spectrophotometer (Multiskan GO 1.00.38, Thermo Scientific, Hudson, NH, USA). Standards of 0.1–2 μg phosphorus (NaH_2_PO_4_⋅2H_2_O, 100 μg of phosphorus/mL) underwent the same treatment as the lipid extract, excluding the hydrolysis step. The conversion factor of 25 (775/31) was used to estimate the PL amount.

### 2.5. Quantification of Glycolipids

Glycolipids present in the lipid extract were quantified using the orcinol colorimetric method, as previously described [13]. Briefly, the sugar amount present in 25 µL of lipid extract reacted with 1 mL of orcinol solution (0.2% in 70% H_2_SO_4_) at 80 °C for 20 min and the absorbance was measured at 505 nm using a UV/vis spectrophotometer (Multiskan GO 1.00.38, Thermo Scientific, Hudson, NH, USA). D-Glucose standards of 2–50 μg (D-glucose, 2.0 mg/mL) were prepared and underwent the same treatment as the lipid extract samples. The conversion factor of 2.8 (100/35) was used to estimate the GL amount.

### 2.6. Quantification of Triglycerides

Triglycerides were quantified with a commercial kit (Liquick Cor-TG 30 (PZ CORMANY S.A., Łomianki, Poland)) according to the manufacturer’s instructions. Liposomes of dried lipid extract (4 mg/mL) were prepared in 5 mM ammonium bicarbonate buffer (pH 7.4), as previously described [3].

### 2.7. Quantification of Pigments

Total chlorophyll and carotenoids in lipid extracts (100 μg in 500 μL of methanol) were estimated by measuring the absorbance at 678 nm for chlorophyll and 440 nm for carotenoids using standard calibration curves of each pigment (chlorophyll *a* and β-carotene, respectively). Results were expressed as the sum of total chlorophyll and carotenoids.

### 2.8. C18 Liquid Chromatography-Mass Spectrometry (C18 LC-MS)

The lipid extracts were analyzed using C18 reverse-phase liquid chromatography mass spectrometry (C18 RP-LC-MS) on an Ultimate 3000 Dionex ultra high-performance liquid chromatography (UHPLC) system (Thermo Fisher Scientific, Bremen, Germany) with an autosampler coupled to the Q-Exactive^®^ hybrid quadrupole Orbitrap mass spectrometer (Thermo Fisher, Scientific, Bremer, Germany) [14]. The solvent system included two mobile phases: mobile phase A (water/acetonitrile (40/60%), 10 mM ammonium formate, and 0.1% formic acid) and mobile phase B (isopropanol/acetonitrile (90/10%), 10 mM ammonium formate, and 0.1% formic acid). The following gradient was applied: 32% B at 0 min, 45% B at 1.5 min, 52% B at 4 mi, 58% B at 5 min, 66% B at 8 min, 70% B at 11 min, 85% at 14 min, 97% at 18 min, 97% B at 25 min, 32% B at 25.01 min, and 32% B at 33 min. A volume of 5 µL, containing 40 µg of lipid extract diluted in dichloromethane (2 µg/µL), 72 µL of a solvent system (50% isopropanol and 50% of methanol), and 8 µL of a phospholipid standard mixture, was loaded onto an Ascentis^®^ Express C18 column (Sigma-Aldrich^®^, 2.1 × 150 mm, 2.7 µm) at 50 °C and at a flow rate of 260 µL min^−1^. The mass spectrometer was operated using positive/negative switching toggles between positive (electrospray voltage 3.0 kV) and negative (electrospray voltage −2.7 kV) ion modes, using a sheath gas flow of 35 U and a capillary temperature of 320 °C. Data were acquired in full scan mode with a high resolution of 70,000, an automatic gain control (AGC) target of 3 × 10^6^, in an *m/z* range of 300–1600, 2 microscans, and a maximum inject time (IT) of 100 ms. The tandem mass spectra (MS/MS) were obtained with a resolution of 17,500, an AGC target of 1 × 10^5^, 1 microscan, and a maximum IT of 100 ms. The cycles consisted of one full-scan mass spectrum and ten data-dependent MS/MS scans, which were repeated continuously throughout the experiments with a dynamic exclusion of 30 s and an intensity threshold of 8 × 10^4^. Normalized collision energy TM (CE) ranged between 20, 24, and 28 eV in the negative mode and 25 and 30 eV in the positive mode. Data acquisition was carried out using the Xcalibur data system (V3.3, Thermo Fisher Scientific, Bremen, Germany). The lipid species were manually confirmed based on the fragmentation pattern of each lipid class, as previously reported [15]. Phosphatidylcholine (PC), lyso PC (LPC), diacylglyceryl-*N*,*N*,*N*-trimethyl homoserine (DGTS) and its lyso form monoacylglyceryl-*N*,*N*,*N*-trimethyl homoserine (MGTS), ceramides (Cer), and ceramide phosphoinositol (PI-Cer) were analyzed in the positive ion mode and identified in MS spectra as [M+H]^+^ ions. In the MS/MS spectra of the [M+H]^+^ ions, LPC and PC were identified by the presence of the fragment ion at *m*/*z* 184.1, corresponding to the phosphocholine polar head. The DGTS and MGTS were confirmed by the presence of the fragment ion at *m*/*z* 236.1, formed due to the loss of the fatty acyl chain as ketene (-R1=C=O/-R2=C=O), and observed on the LC-MS/MS spectra of the [M+H]^+^ ions. The fatty acyl composition for these betaine classes was assigned by the neutral loss of one or two fatty acyl chains as ketene (-R=C=O) and as acid (-RCOOH). In the case of both Cer and PI-Cer, the sphingoid backbone (SB) was assigned due to the presence of fragment ions corresponding to [SB-H_2_O]^+^ and [SB-2H_2_O]^+^ ions, while the fatty acyl chain composition was obtained, whenever possible, by the mass difference calculation between SB and the ceramide precursor ion. Although digalactosyl diacylglycerol (DGDG), digalactosyl monoacylglycerol (DGMG), monogalactosyl diacylglycerol (MGDG), monogalactosyl monoacylglycerol (MGMG), triglycerides (TGs), and diglycerides (DGs) were also analyzed in positive ion mode, these lipid classes were identified in MS as [M+NH_4_]^+^ ions. MGDG and MGMG species were identified by the typical neutral loss of 197 Da, corresponding to the neutral loss of NH_3_ (−17 Da) plus the galactosyl moiety (−180 Da), while DGDG and DGMG were identified due to the neutral loss of 359 Da, which corresponds to the neutral loss of NH_3_ (−17 Da) plus the digalactosyl moiety (−342 Da). The fatty acyl chain composition was attributed to the presence of acylium ions of FA plus 74 Da ([RCO + 74]^+^). TG and DG were identified by the neutral loss of NH_3_ (−17 Da) combined with the neutral loss of the FA as acid derivatives (-RCOOH + NH_3_).

Phosphatidylglycerol (PG), phosphatidylinositol (PI), sulfoquinovosyl diacylglycerol (SQDG), and its lyso form sulfoquinovosyl monoacylglycerol (SQMG) were analyzed in negative ion mode and identified as [M-H]^−^ ions in MS spectra. In MS/MS spectra, SQDG and SQMG were assigned due to the presence of the fragment ion at *m*/*z* 225.0, corresponding to the anion of the sulfoquinovosyl polar head group. The fatty acyl chain composition was determined by the neutral loss of each fatty acid as acid (-RCOOH) and, whenever possible, by the presence of the carboxylate anions of fatty acyl chains (RCOO^−^). The lipid molecular species belonging to the PG class were assigned due to the presence of the fragment ions at *m*/*z* 153.0 (glycerol phosphate anion—H_2_O), 171.0 (glycerol phosphate anion), and 227.0 (glycerolphosphate glycerol—H_2_O), while the lipid molecular species belonging to the PI class were identified by the observation of the fragment ions at *m*/*z* 241.0 (inosiyol-1,2-cyclic phosphate anion), 223.0 (241—H_2_O), 297.0 (combined loss of fatty acyl chains as acid (RCOOH)), and 315.0 (combined loss of fatty acyl chains as ketene (R=C=O) and acid (RCOOH)). In PG and PI, the fatty acyl chain composition was confirmed due to the presence of the carboxylate anions of fatty acyl chains (RCOO^−^).

Validated species were integrated using MZmine v2.53 software and normalized by calculating the ratio against a selected internal standard (Table S1) with the closest retention time. Peak area integration was adjusted and manually edited if necessary (Table S2). The relative abundance (%) of each lipid species within each class was calculated as described before [16].

### 2.9. Fatty Acid Analysis by GC-MS

The fatty acid methyl esters (FAMEs) were prepared from dried lipid extract by alkaline trans-esterification, as previously described [12], and analyzed by gas chromatography coupled with mass spectrometry (GC–MS). Briefly, 2 µL of a hexane solution containing FAMEs and 1.0 µg/mL of methyl nonadecanoate, as an internal standard, were injected onto the GC-MS instrument. Data acquired were analyzed using Agilent MassHunter Qualitative Analysis 10.0 software, which together with the NIST library and by comparing the retention times with those of commercial standards (Supelco 37 Component Fame Mix, Sigma-Aldrich, St. Louis, MO, USA), allowed the identification of FAs.

(2) $$AI=\frac{\left[C12:0+\left(4\times C14:0\right)+C16:0\right]}{\left[\sum MUFA+\sum \left(n-6\right)+\sum \left(n-3\right)\right]},$$

(3) $$TI=\frac{\left[C14:0+C16:0+C18:0\right]}{\left[\left(0.5\times MUFA\right)+(0.5\times \sum \left(n-6\right))+\left(3\times \sum \left(n-3\right)\right)+\left(\frac{\sum \left(n-3\right)}{\sum \left(n-6\right)}\right)\right]},$$

(4) $$h/H=\frac{\left[cis-C18:1+\sum PUFA\right]}{\left[C12:0+C14:0+C16:0\right]},$$

### 2.10. Anti-Inflammatory Activity

The anti-inflammatory activity was determined using the commercial cyclooxygenase-2 (COX-2) inhibitory screening assay kit (Cayman test kit-701080 (Cayman Chemical Company, Ann Arbor, MI, USA)), according to the manufacturer’s instructions. The dried lipid extract was dissolved in DMSO (10 µL) to obtain a concentration of 100 µg/mL and the amount of prostaglandin F2α produced was measured by spectrophotometry (415 nm) using a UV/vis spectrophotometer (Multiskan GO 1.00.38, Thermo Scientific, Hudson, NH, USA). The results were expressed as a percentage of inhibited COX-2 activity.

### 2.11. Antioxidant Activity

The antioxidant capacity was evaluated against DPPH^•^ and ABTS^•+^ radicals, as described before [13]. The dried lipid extract was dissolved in ethanol (25, 125, and 250 µg/mL) and mixed with a working solution of DPPH^•^ or ABTS^•+^ in ethanol (abs ~0.9). The absorbance of the samples was measured at 517 nm or 734 nm for DPPH^•^ or ABTS^•+^, respectively, every 5 min for a total of 120 min of incubation, using a UV/vis spectrophotometer (Multiskan GO 1.00.38, Thermo Scientific, Hudson, NH, USA). Control samples were also prepared by replacing the DPPH• or ABTS•+ with ethanol. DPPH^•^ and ABTS^•+^ stability in ethanol was monitored. All measurements were performed in triplicate. Trolox standard solutions (between 10 and 75 μM in ethanol) were also prepared and underwent the same treatment as the lipid extract samples. Results were expressed as a percentage of inhibition of DPPH^●^ (IC_15_) or ABTS^•+^ (IC_50_) and in Trolox equivalents (TE, µmol Trolox/g LE).

(5) $$Inhibition\;\%=\frac{{Abs}_{radical}-({Abs}_{radical}-{Abs}_{control})}{{Abs}_{radical}}\times 100,$$

(6) $$TE\left(\frac{µmol}{g}\right)=\frac{{IC}_{15}\;or\;{IC}_{50}\;Trolox\left(\frac{µmol}{L}\right)}{{IC}_{15}\;or\;{IC}_{50}\;of\;samples\left(\frac{µg}{mL}\right)}\times 1000,$$

### 2.12. Antidiabetic Activity—α-Glucosidase Inhibition Assay

The capacity to inhibit α-glucosidase activity was determined using a colorimetric-based quantitative method, as described before [17]. Liposomes of dried lipid extract (25 and 125 µg/mL) were prepared in 0.1 M phosphate buffer (pH 6.9), as previously described [3]. In a 96-well plate, 50 µL of liposomes and 100 µL of 0.1 M phosphate buffer (pH 6.9) containing α-glucosidase solution (1.0 U/mL) were incubated at 25 °C for 10 min. Then, 50 µL of a 5 mM ρ-nitrophenyl-α-D-glucopyranoside solution prepared in 0.1 M phosphate buffer (pH 6.9) was added to each well. The plate was incubated at 25 °C for another 5 min, and absorbance readings were recorded at 405 nm using a UV/vis spectrophotometer (Multiskan GO 1.00.38, Thermo Scientific, Hudson, NH, USA). Negative and positive controls were prepared by replacing 50 µL of the sample with potassium phosphate buffer and acarbose (10 mg/mL), respectively.

### 2.13. Statistical Analysis

The results are expressed as averages ± standard deviation (SD), and comparisons between groups were made using one-way ANOVA followed by Tukey’s multiple comparison test. GraphPad (version 8.0.2) was used for all comparisons and, when *p* < 0.05, the differences were considered significant.

## 3. Results and Discussion

### 3.1. Lipid Content

The total lipid content of *D. salina* was 6.8 ± 2.1 (% DW), as reported in previous studies [18,19,20]. It also followed the same trend as the lipid content reported for *Spirulina* sp., *Chlorella vulgaris*, and *Tetraselmis chui*, which ranged between 6.5 and 10.7% using the same extraction method. Therefore, the lipid content in *D. salina* is within the range of other microalgae approved as food [12].

**Figure 1:**
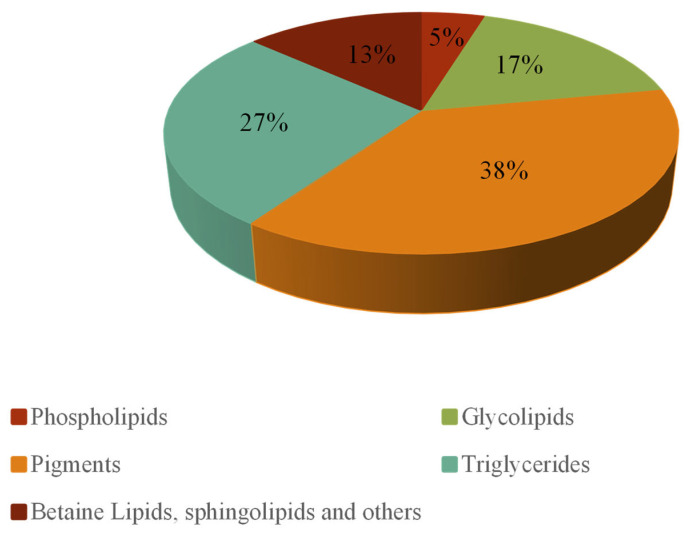
Composition of *Dunaliella salina* lipid extract (LE), expressed as a percentage (%).

The PL and GL content accounted, respectively, for 47.48 ± 6.21 and 138.58 ± 46.39 µg/mg of the total lipid content and both represented 22% of the total lipids (Figure 1), similar to what is reported in the literature [18,21]. Additionally, TG represented 27% of the total lipids, while pigments represented 38%, predominantly consisting of carotenoids, particularly β-carotene, while chlorophylls were present in very low amounts. The remaining 13% included betaine lipids, sphingolipids, and other neutral lipids.

### 3.2. Lipidome Profiling

**Figure 2:**
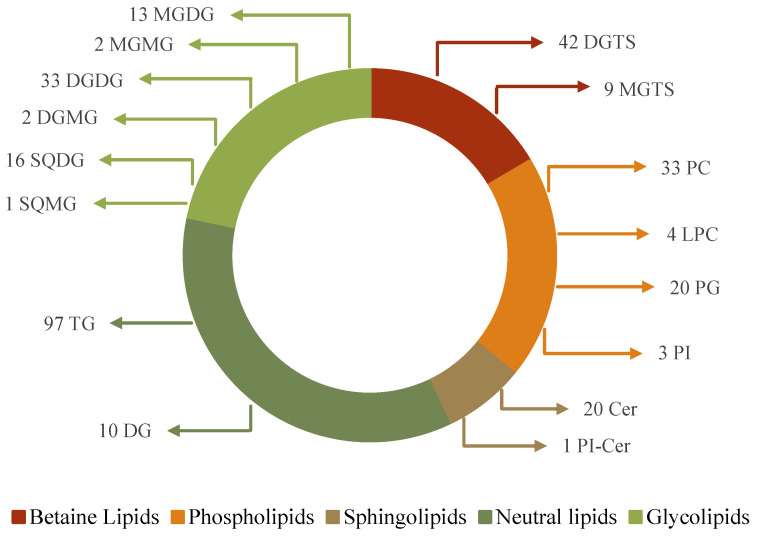
Number of lipid molecular species identified in *Dunaliella salina* lipid extract (LE) by C18 reverse-phase liquid chromatography-mass spectrometry (C18 RP-LC-MS), distributed by the classes of glycolipids: monogalactosyl diacylglycerol (MGDG), monogalactosyl monoacylglycerol (MGMG), digalactosyl diacylglycerol (DGDG), digalactosyl monoacylglycerol (DGMG), sulfoquinovosyl diacylglycerol (SQDG), and sulfoquinovosyl monoacylglycerol (SQMG); betaine lipids: diacylglyceryl-*N*,*N*,*N*-trimethyl homoserine (DGTS) and monoacylglyceryl-*N*,*N*,*N*-trimethyl homoserine (MGTS); phospholipids: phosphatidylglycerol (PG), phosphatidylinositol (PI), phosphatidylcholine (PC), and lysophosphatidylcholine (LPC); sphingolipids: ceramide (Cer) and inositolphosphoceramide (PI-Cer); neutral lipids: triglycerides (TGs) and diglycerides (DGs); and fatty acids (FAs).

In total, 306 lipid molecular species were identified (Figure 2, Table 1 and Table S2–S7), belonging to the following classes: PL (60 lipid species), GL (67 lipid species), betaine lipids (51 lipid species), sphingolipids (21 lipid species), and neutral lipids (107 lipid species). The PL classes identified were phosphatidylcholine (PC), lyso PC (LPC), phosphatidylglycerol (PG), and phosphatidylinositol (PI). The most abundant PL species were PC 36:2 (PC 18:1/18:1) and PG 34:2 (PG 16:0_18:2), which corresponded to 20% of the total content of PL classes PC and PG, respectively, and PI 34:1 (PI 16:0_18:1), which corresponded to 76% of the total content of identified PI lipid species (Table 1, Figures S1 and S6). A considerable number of PL species, including the most abundant ones, had omega-3 PUFA and FA 18:1. The GL classes identified included monogalactosyldiacylglycerol (MGDG), monogalactosyl monoacylglycerol (MGMG), digalactosyl diacylglycerol (DGDG), digalactosyl monoacylglycerol (DGMG), sulfoquinovosyl diacylglycerol (SQDG), and sulfoquinovosyl monoacylglycerol (SQMG). Several GL species with omega-3 PUFA and FA 18:1, such as MGDG 34:7 and DGDG 34:1, were the lipid species with higher relative abundance in each respective class (Figures S2 and S6). Betaine lipids identified were diacylglyceril-*N*,*N*,*N*-trimethylhomoserine (DGTS) and monoacylglyceryl-*N*,*N*,*N*-trimethylhomoserine (MGTS), which had the highest number of lipid species with FA 18:1 and FA 18:3 (Figures S3 and S6). Some sphingolipid species were identified, with Cer 18:2;O/17:0 being the most abundant in the respective class and PI-Cer 34:1;O2 being the only one identified in its class (Figures S4 and S6). Neutral lipids, including triglycerides (TG) and diglycerides (DG), had the largest number of lipid molecular species. The lipid species TG 16:0/16:0/18:1 and DG 16:0/18:3 were in higher abundance in their respective classes (Figures S5 and S6). Interestingly, almost half of the TG species identified had esterified FA 18:1, similar to TG from olive oil [22].

Despite the evident lack of knowledge on the lipid signature of *D. salina*, especially at the molecular level, Vanitha and co-authors were able to identify other lipid classes not reported here by only using thin layer chromatography, namely monoglycerides (MGs), sterols, phosphatidylethanolamine (PE), and phosphatidic acid (PA) [23]. In addition, five DGs, three MGDGs, and five DGDGs, identified using HPLC to separate lipid species and a UV detector to identify the FA composition, were also identified in previous studies [24,25]. In a previous study, using a low-resolution linear ion-trap mass spectrometer, several lipid species were identified, not from *D. salina* but from *Dunaliella tertiolecta*, including those belonging to the PI, DGDG, MGDG, SQDG, DGTS, and MGTS classes, which were also identified in the present study [26]. The common lipid species included PI 34:1 (16:0_18:1), DGDG 34:7 (16:4_18:3), DGDG 34:6 (16:3_18:3), DGDG 34:5 (16:2_18:3), DGDG 34:4 (16:1_18:3), DGDG 34:2 (16:0_18:2), DGDG 34:1 (16:0_18:1), MGDG 34:7 (16:4_18:3), MGDG 34:6 (16:4_18:2 and 16:3_18:3), SQDG 32:0, SQDG 34:3, SQDG 34:2, SQDG 34:1, DGTS 34:4 (16:0_18:4), DGTS 34:3 (16:0_18:3), DGTS 34:2 (16:0_18:2), DGTS 34:1 (16:0_18:1), DGTS 35:3 (17:0_18:3), DGTS 36:6 (18:3/18:3), DGTS 36:5 (18:2_18:3), DGTS 36:4 (18:2/18:2 and 18:1_18:3), DGTS 36:3 (18:1_18:2), DGTS 36:2 (18:1/18:1), DGTS 37:3 (18:2_19:1), MGTS 16:0, MGTS 18:3, and MGTS 18:2. Nevertheless, with the modern LC-MS/MS lipidomics approach employed here, we were able to deepen our knowledge of the *D. salina* lipidome. Polar lipids from olive fruits (*Olea europaea* L.) also included lipid molecular species from phospholipids, glycolipids, sphingolipids, and betaine lipids. Several lipid molecular species of PC, LPC, PG, MGDG, DGDG, DGMG, and DGTS were esterified with FA 18:1 *n*-9, as well as PUFAs 18:2 *n*-6 and 18:3 *n*-3. In fact, the most abundant lipid species of PC and PG classes found in olives fruits were PC 36:2 and PG 36:2, as in the lipid profile of *D. salina*. This composition highlights the similarity between the polar lipid profiles of *D. salina* and olive fruits. Interestingly, PI, SQDG, SQMG, and MGMG classes were not reported in the lipid signature of olives fruits, while phosphatidylethanolamine (PE) and lyso PE were not identified in this microalga. The sphingolipid profile also showed significant differences. The sphingolipid classes identified in *D. salina* were Cer and PI-Cer, while sphingomyelins and hexosylceramides (HexCer) were found in olive fruits [27]. A similar lipid composition was also observed between *D. salina* and olive (*Olea europaea* cv. *Galega vulgar*) fruit seeds, except for certain lipid classes. Specifically, betaine lipids, PI, SQDG, SQMG, MGMG, DGMG, and PI-Cer classes were not detected in olive seeds, while PE, LPE, HexCer, and acyl sterol glycosides were not identified in *D. salina* [28]. In the lipid signature of olives fruits, FA 18:1 *n*-9, FA 18:2 *n*-6, and FA 18:3 *n*-3 were once more the most representative FAs in polar lipid molecular species, as is the case in *D. salina*. Hydroxylated polar lipid species, namely PC, were also identified in olive fruits [27] and seeds [28]. Many TG species identified in the lipid profile of *D. salina* were esterified with FA 18:1 *n*-9 and essential PUFAs (18:2 *n*-6 and 18:3 *n*-3), as well as with odd-chain FAs ranging from FA13:0 to FA 27:0 and odd-chain MUFAs (FA 15:1, FA 17:1, and FA 19:1) and PUFAs (FA 19:2), and these FAs were also found esterified in TGs from olive fruits (*Olea europaea* L.) [27] and pistachio nuts [29]. In addition, the lipid signature of *D. salina* is similar to that of avocado, a globally important fruit crop with rising consumption due to the recognition of its nutritional and health benefits. Common lipid classes include PC, PI, DGDG, MGDG, TG, and DG, comprising several lipid molecular species esterified with FA 18:1 and essential PUFAs. However, avocado also contains the polar lipid classes PE, phosphatidylserine (PS), and phosphatidic acid (PA) [30]. This highlights the similar lipid signature of *D. salina* with common plant-based foods. However, when comparing the lipid profile of *D. salina* with that of animal-based foods, such as fish and other edible aquatic animals, more pronounced differences emerge. Notably, the lipid composition of animal-based seafood is characterized by the presence of PL classes, including PS and cardiolipin, as well as sphingolipid classes such as ceramide phosphoethanolamine, ceramides aminoethylphosphonate, and *N*-methyl ceramide aminoethylphosphonate, along with several ether phospholipid species, which are all less reported in plant- and algae-based foods. However, animal-based foods lack chloroplast-specific lipid classes (DGDG, MGDG, SQDG, and their lyso forms) [15,31].

Interestingly, some polar lipid species herein identified, such as LPC 16:0, LPC 16:1, LPC 18:2, LPC 18:3 [32], PG 34:2 (16:0/18:2) [33], and MGDG 34:7 (16:4/18:3) [34], as well as other algae glycolipids, were previously reported with anti-inflammatory potential, among other biological properties [35]. PL species bearing omega-3 PUFA and FA 18:1, as in *D. salina*, were also reported to have positive effects on reducing the risk of cardiovascular diseases [36].

PL species can also be used in nano-liposomes and as carriers of drugs, as well as in cosmetics, as natural ingredients. They can also be used as emollients, emulsifiers, and antioxidants, thus being an alternative to synthetic counterparts.

### 3.3. Fatty Acid Profiling

The FA profile of *D. salina* studied by GC-MS allowed the identification of 19 FAs (Table 2). The saturated FA 16:0 was the most abundant (43.35 ± 1.67%), followed by FA 18:1 *n*-9 and FA 18:3 *n*-3 (14.66 ± 1.09 and 13.55 ± 1.58%, respectively), in accordance with previous studies [7,8,19]. *Dunaliella salina* has a high amount of FA 18:1 *n*-9, which has been associated with the cardioprotective effects of the Mediterranean diet, as in the case of extra virgin olive oil [22]. PUFAs represented almost one-fourth of the total FAs (~27%) of *D. salina* and omega-3 PUFA accounted for 17.60 ± 2.02%. Among omega-3 PUFAs, FA 18:3 *n*-3 showed the highest abundance (13.55 ± 1.58%). This is an essential FA in the human diet as it is metabolized to FA 20:5 *n*-3 (eicosapentaenoic acid, EPA) and FA 22:6 *n*-3 (docosahexaenoic acid, DHA), which play a central role in proper tissue functioning [37].

As mentioned, omega-3 PUFAs are well-recognized for their beneficial effects on health status and help to prevent NCDs such as atherosclerosis and cardiovascular diseases [1]. Thus, lipid quality indexes were calculated from the FA profile, namely the *n*-6/*n*-3 ratio, atherogenic index (AI), thrombogenic index (TI), and hypocholesterolemic/hypercholesterolemic (h/H) ratio, to estimate the nutritional value and health benefits of *D. salina* lipids. The *n*-6/*n*-3 ratio was lower than 1 (0.49 ± 0.04), which can be associated with the prevention of cardiovascular complications. The lower TI (0.64 ± 0.07) and AI (0.93 ± 0.06) indicate a protective effect in reducing blood clots and atherogenic plaque formation, respectively [38]. The h/H obtained was 0.88 ± 0.05, and the higher the h/H values, the more beneficial effect they have by lowering cholesterol levels. The AI, TI, and h/H values obtained for *D. salina* were all within the range of those obtained for other microalgae approved as foods and food supplements, such as *Spirulina* sp. (AI of 0.7, TI of 1.6, and h/H of 0.6) [12] and *Chlorella vulgaris* (AI of 0.2–0.42, TI of 0.21, and h/H of 2.04–2.8) [12,39], and *Nannochloropsis oculata* (AI of 0.63, TI of 0.22, and h/H of 1.44) and *Microchloropsis gaditana* (AI of 1.70, TI of 3.82, and h/H of 0.12) [39], respectively. *Dunaliella salina* showed lipid quality indices comparable to those of fish, shrimp, and shellfish hake (AI: 0.26–1.41; TI: 0.09–0.87; h/H: 0.21–4.75) [40], as well as of a variety of fish oils (AI: 0.13–1.48; TI: 0.11–1.16; h/H: 0.67–8.03) [41]. Despite the fact that AI and TI indices for *D. salina* were higher than those reported for olive oil (AI: 0.16 and TI: 0.19), both were lower than those reported for palm oil (AI and TI of 1.88) [42]. Therefore, *D. salina* can be a promising alternative to fish, fish oil, and palm oil, reinforcing its potential as a sustainable source of healthy lipids and minimizing the environmental and economic impacts of overfishing and overexploitation of arable land and deforestation required for palm oil plantations.

Overall, the *D. salina* lipidome is rich in FA 18:1 *n*-9 and FA 18:3 *n*-3, being a promising alternative as a natural source of novel functional ingredients with healthy effects and for the prevention of oxidative stress and inflammatory-related diseases, as well as dyslipidemia. To add value to *D. salina* biomass, we also explored the bioactive potential of its lipid extract.

### 3.4. Bioactive Properties of D. salina Lipids

The lipid extract of *D. salina* showed anti-inflammatory activity at 100 µg/mL, inhibiting COX-2 activity by 35.68 ± 5.16% (Table 3), which is similar to that reported for *Chlorella vulgaris* [14], a microalga that has been used as a food ingredient, food supplement, and food additive [43,44]. To the best of our knowledge, only one study showed the inhibitory effect of *D. salina n*-3 PUFA concentrate (20 µg/mL) on COX-2 expression rather than enzymatic activity [45]. GL from *D. salina* may contribute to this effect since GL from microalgae has been correlated with anti-inflammatory potential [46]. Interestingly, it was reported that MGDG 34:7, also identified in *D. salina*, inhibited ^•^NO levels in LPS-stimulated RAW 264.7 macrophages co-treated with the methanolic extract of *Tetraselmis chui* through downregulation of iNOS [34]. Moreover, four lyso forms of PC, namely 16:0, LPC 16:1, LPC 18:2, and LPC 18:3, also identified in *D. salina* were shown to inhibit TNF-α release in LPS-stimulated THP-1 cells co-treated with fractionated lipid extracts of the diatom *Cylindrotheca clostridium* [32]. These lipid species may contribute to the anti-inflammatory potential of the lipid extract of *D. salina*.

The DPPH^•^ and ABTS^•+^ radical scavenging assays were used to evaluate the antioxidant potential of the lipid extract of *D. salina*, which showed higher scavenging activity against ABTS^•+^, with an IC_50_ of 81.99 ± 1.14 µg/mL and a TE of 223.97 ± 3.12 µmol Trolox/g LE (Table 3). Similar results were observed for lipid extracts of other microalgae used as food and food supplements [12,14]. However, it was reported that the methanol extract of *D. salina* was able to inhibit DPPH^•^ and ABTS^•+^ by 79 and 50%, respectively, but using a much higher concentration of 80 mg/mL [47], while its chloroform extract inhibited DPPH^•^ by 50% at 1.92 mg/mL [48]. This suggests that the lipid extract of *D. salina* is a potential source of natural antioxidants, with applications in the food industry as ingredients and stabilizers.

**Figure 3:**
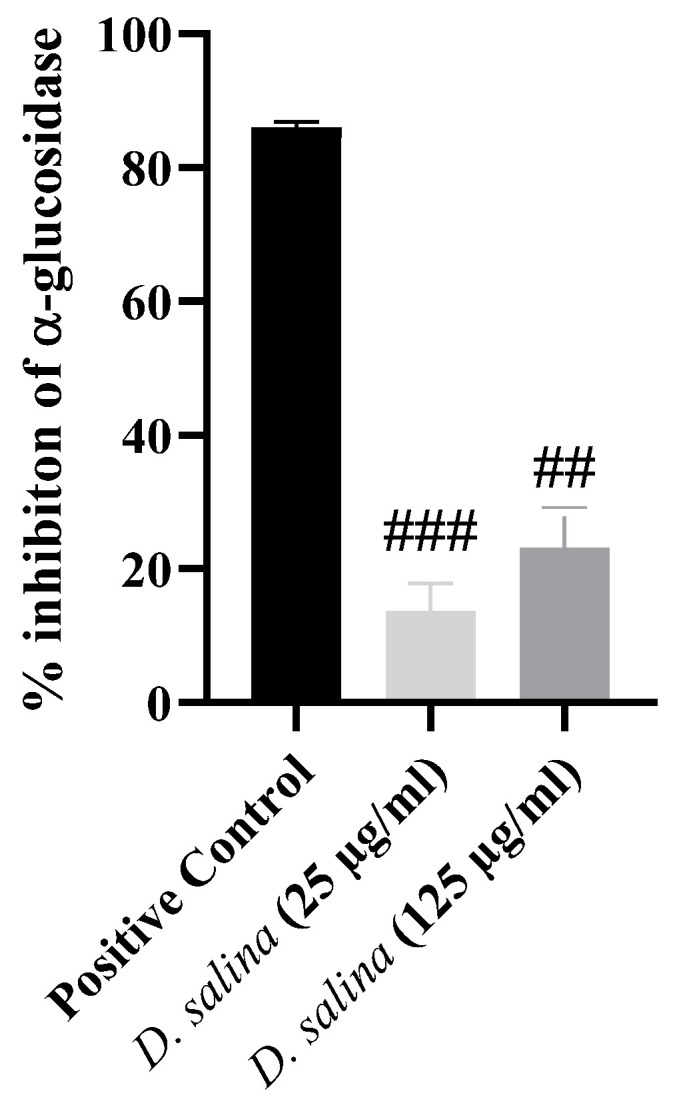
Inhibitory effect of *Dunaliella salina* lipid extract (LE) at 25 and 125 µg/mL on α-glucosidase activity. Values are presented as the average of three assays (*n* = 3) ± standard deviation. Differences with a *q*-value < 0.05 were considered statistically significant. ##: *q* < 0.01; ###: *q* < 0.001.

The inhibitory effect of the lipid extract of *D. salina* on α-glucosidase activity was assayed to evaluate its antidiabetic potential. At 25 and 125 µg/mL, the lipid extract of *D. salina* inhibited enzyme activity by 14 and 23%, respectively (Figure 3). The inhibitory effect of lipids extracted from *D. salina* on α-glucosidase activity has not been reported yet; however, a few compounds present in algae, namely ALA, EPA, DHA, pigments, and polyphenols, have been suggested as displaying antidiabetic activity [49]. With a slightly higher concentration than the one used in this study (200 µg/mL), the lipid extract of *Chlorella pyrenoidosa* exhibited a similar percentage of inhibition of α-glucosidase activity (22.15%) [5]. Additionally, hydrolysates from *Chlorella vulgaris* (30 mg/mL) showed the capability to inhibit enzyme activity by 31% [50]. Overall, the antidiabetic activity displayed by *D. salina* is very similar to that observed for other bioactive compounds extracted from microalgae, such as FA, pigments, and polyphenols.

However, it is important to note that pigments, particularly β-carotene, can also contribute synergistically to the overall observed activity. In fact, β-carotene from *Dunaliella salina* has been reported in the literature to exhibit various biological properties, as reviewed in [51], namely neuroprotective, hepatoprotective, and antiproliferative capacities, as well as anti-inflammatory properties and high antioxidant potential. Indeed, a previous study used a carotenoid-enriched extract from *D. salina* to evaluate its anti-cholinergic, anti-inflammatory, and antioxidant effects using *in chemico* assays, as well as its potential neuroprotective effect against key hallmarks of Alzheimer’s disease (AD) in the human neuron-like SH-SY5Y cell model [52]. Lipidomic changes, including increases in PC, TG, and FA levels and a decrease in PG in cells, along with the possible role exerted by carotenoids and other minor compounds on the cell membrane, were linked to the observed neuroprotective effect of the *D. salina* extract. Another study showed that *D. salina* extracts rich in TGs, FAs, and β-carotene have neuroprotective effects in an Alzheimer’s disease model of *Caenorhabditis elegans* (strain CL4176) [53]. Additionally, *D. salina* powder and extracts rich in β-carotene showed pronounced protective activity against thioacetamide-induced liver fibrosis [54].

Thus, *Dunaliella salina* can be a valuable source of lipids and other bioactive compounds, such as β-carotene, that can be explored as new functional ingredients to be used in the non-pharmacological management of diabetes, metabolic syndrome, obesity, and other health conditions, as well as for different biotechnological applications and in the food and nutraceutical industries.

## 4. Conclusions

This is the first report on the full characterization of the *D. salina* lipidome, as well as the anti-inflammatory, antioxidant, and antidiabetic potential of its lipids. A considerable number of identified polar lipid species contained esterified OA and ALA, with omega-3 PUFAs accounting for 17% of total FAs. The lipid extract of *D. salina* showed anti-inflammatory activity through inhibiting COX-2 activity, antioxidant potential through scavenging DPPH^•^ and ABTS^•+^, and antidiabetic activity by inhibiting α-glucosidase activity. Overall, these results highlight the potential of this microalga to be used as a food ingredient or in the nutraceutical and cosmeceutical industries.

## Figures and Tables

**Table 1:** Classes of lipids identified in *Dunaliella salina* lipid extract (LE). The number of lipid molecular species identified in each class, as well as the number of lipid molecular species with FA 18:3 and FA 18:1 and the top 3 most abundant lipid molecular species, are also shown.

**Phospholipid**	**Total Number of Lipid Molecular Species**	**Number of Lipid Molecular Species with FA 18:3**	**Number of Lipid Molecular Species with FA 18:1**	**Most Abundant Molecular Species**
PC	33	1	3	PC 18:1/18:1; PC 16:0_18:1; PC 16:0_18:2
LPC	4	1	0	LPC 16:0; LPC 18:2; LPC 18:3
PG	20	2	6	PG 16:0_18:2; PG 18:1/18:1; PG 16:0_18:1
PI	3	1	1	PI 16:0_18:1; PI 16:0_18:2; PI 16:0_18:3
**Glycolipid**	**Total Number of Lipid Molecular Species**	**Number of Lipid Molecular Species with FA 18:3**	**Number of Lipid Molecular Species with FA 18:1**	**Most Abundant Molecular Species**
MGDG	13	4	4	MGDG 16:4_18:3; MGDG 16:4_18:2; MGDG 16:0_18:1
MGMG	2	1	0	MGMG 18:3; MGMG 16:0
DGDG	33	8	7	DGDG 16:0_18:1; DGDG 16:1_18:2; DGDG 16:1_18:1
DGMG	2	1	0	DGMG 16:0; DGMG 18:3
SQDG	16	0	0	SQDG 32:0; SQDG 34:3; SQDG 34:1
SQMG	1	0	0	SQMG 16:0
**Betaine Lipids**	**Total Number of Lipid Molecular Species**	**Number of Lipid Molecular Species with FA 18:3**	**Number of Lipid Molecular Species with FA 18:1**	**Most Abundant Molecular Species**
DGTS	42	14	12	DGTS 16:1_18:2; DGTS16:0_18:2; DGTS 16:0_18:1
MGTS	9	1	1	MGTS 18:2
**Sphingolipids**	**Total Number of Lipid Molecular Species**	**Number of Lipid Molecular Species with FA 18:3**	**Number of Lipid Molecular Species with FA 18:1**	**Most Abundant Molecular Species**
Cer	20	0	1	Cer 18:2;O/17:0; Cer 22:0;O3/26:0; Cer 35:0;O2_3
PI-Cer	1	0	0	PI-Cer 20:1;O2/14:0
**Neutral Lipids**	**Total Number of Lipid Molecular Species**	**Number of Lipid Molecular Species with FA 18:3**	**Number of Lipid Molecular Species with FA 18:1**	**Most Abundant Molecular Species**
TG	97	21	43	TG 16:0_16:0_18:1; TG 16:0_18:1_18:1; TG 16:0_16:0_18:2
DG	10	2	3	DG 16:0_18:3;O2; DG 16:0_18:3; DG 18:1/18:1

Abbreviations: PC: phosphatidylcholine; LPC: lyso phosphatidylcholine; PG: phosphatidylglycerol; PI: phosphatidylinositol; MGDG: monogalactosyl diacylglycerol; MGMG: monogalatosyl monoacylglycerol; DGDG: digalactosyl diacylglycerol; DGMG: digalactosyl monoacylglycerol; SQDG: sulfoquinovosyl diacylglycerol; SQMG: sulfoquinovosyl monoacylglycerol; DGTS: diacylglyceryl-*N*,*N*,*N*-trimethyl homoserine; MGTS: monoacylglyceryl-*N*,*N*,*N*-trimethyl homoserine; Cer: ceramide; PI-Cer: inositolphosphoceramide; TG: triglycerides; DG: diglycerides.

**Table 2:** Fatty acid (FA) profile of *Dunaliella salina* determined by gas chromatography-mass spectrometry (GC-MS) after base-catalyzed transmethylation of the lipid extracts. Data are expressed as µg of FA by mg of biomass and as relative abundance (%) ± SD (*n* = 5).

Fatty Acid	µg FA/mg Biomass	Relative Abundance (%)
FA 16:0	8.13 ± 0.77	43.35 ± 1.67
FA 16:1	0.13 ± 0.01	0.69 ± 0.06
FA 16:1 *n*-7	0.27 ± 0.02	1.43 ± 0.12
FA 16:1 *n*-9	0.17 ± 0.03	0.89 ± 0.07
FA 16:2 *n*-6	0.21 ± 0.02	1.09 ± 0.02
FA 16:3 *n*-6	0.09 ± 0.01	0.49 ± 0.01
FA 16:3 *n*-3	0.28 ± 0.04	1.47 ± 0.09
FA 16:4 *n*-3	0.49 ± 0.10	2.59 ± 0.35
FA 17:0	0.04 ± 0.01	0.23 ± 0.04
FA 18:0	1.13 ± 0.32	5.93 ± 1.16
FA 18:1 *n*-9	2.74 ± 0.22	14.66 ± 1.09
FA 18:1 *n*-7	0.42 ± 0.04	2.27 ± 0.23
FA 18:2 *n*-8	0.14 ± 0.02	0.76 ± 0.01
FA 18:2 *n*-6	1.16 ± 0.12	6.21 ± 0.26
FA 18:3 *n*-6	0.15 ± 0.02	0.79 ± 0.07
FA 18:3 *n*-3	2.55 ± 0.47	13.55 ± 1.58
FA 20:0	0.21 ± 0.03	1.16 ± 0.26
FA 22:0	0.22 ± 0.03	1.20 ± 0.26
FA 24:0	0.23 ± 0.02	1.25 ± 0.20
Σ SFA	9.97 ± 0.98	53.11 ± 1.26
Σ MUFA	3.73 ± 0.30	19.95 ± 1.43
Σ PUFA	5.07 ± 0.79	26.94 ± 2.35
Σ PUFA *n*-3	3.31 ± 0.61	17.60 ± 2.02
Σ PUFA *n*-6	1.61 ± 0.17	8.57 ± 0.34
*n*-6/*n*-3	0.49 ± 0.04	
AI	0.93 ± 0.06	
TI	0.64 ± 0.07	
h/H	0.88 ± 0.05	

Abbreviations: Σ SFA: the sum of saturated fatty acids; Σ MUFA: the sum of monosaturated fatty acids; Σ PUFA: the sum of polyunsaturated fatty acids; AI: atherogenic index; TI: thrombogenic index.

**Table 3:** Percentage of inhibition of cyclooxygenase-2 (COX-2) activity by *Dunaliella salina* lipid extract (LE) at 100 µg/mL. Concentration of LE (µg/mL) that inhibited 15% and 50% of DPPH^•^ and ABTS^•+^ radicals, respectively, as well as the Trolox equivalents (TE; µmol/g). Values are presented as the average of three assays (*n* = 3) ± standard deviation.

**Anti-Inflammatory Activity**	**COX-2 Assay**
% inhibition	35.68 ± 5.16
**Antioxidant Activity**	**DPPH^•^ Assay**
TE	54.59 ± 4.32
IC_15_	153.65 ±12.17
**Antioxidant Activity**	**ABTS^•+^ Assay**
TE	223.97 ± 3.12
IC_50_	81.99 ± 1.14

## Data Availability

The original contributions presented in the study are included in the article/Supplementary Materials, further inquiries can be directed to the corresponding author.

## Supplementary Materials

The following supporting information can be downloaded at: https://www.mdpi.com/article/10.3390/foods13203321/s1, Figure S1: Representative total ion chromatogram (TIC) from C18 reversed-phase liquid chromatography-mass spectrometry (C18 RP-LC-MS) analysis of *Dunaliella salina* lipid extract, shown in positive (a) and negative (b) ion modes. Retention time for the elution of the different lipid classes identified in D. salina is also highlighted. Abbreviations: ceramide (Cer), ceramide phosphoinositol (PI-Cer), diacylglyceryl-*N*,*N*,*N*-trimethyl homoserine (DGTS), digalactosyl diacylglycerol (DGDG), digalactosyl monoacylglycerol (DGMG), diglyceride (DG), monoacylglyceryl-*N*,*N*,*N*-trimethyl homoserine (MGTS), monogalactosyl diacylglycerol (MGDG), monogalactosyl monoacylglycerol (MGMG), lyso phosphatidylcholine (LPC), phosphatidylcholine (PC), phosphatidylglycerol (PG), phosphatidylinositol (PI), sulfoquinovosyl diacylglycerol (SQDG), sulfoquinovosyl monoacylglycerol (SQMG), and triglyceride (TG); Figure S2: The relative percentage (%) of normalized peak area abundances of identified species per phospholipid class of *Dunaliella salina*. PC (a), LPC (b), PG (c), and PI (d); Figure S3: The relative percentage (%) of normalized peak area abundances of identified species per glycolipid class of *Dunaliella salina*. MGDG (a), MGMG (b), DGDG (c), DGMG (d), SQDG (e), and SQMG (f); Figure S4: The relative percentage (%) of normalized peak area abundances of identified species per betaine lipid class of *Dunaliella salina*. DGTS (a) and MGTS (b); Figure S5: The relative percentage (%) of normalized peak area abundances of identified species per sphingolipid class of *Dunaliella salina*. Cer (a) and PI-Cer (b); Figure S6: The relative percentage (%) of normalized peak area abundances of identified species per neutral lipid class of *Dunaliella salina*. TG (a) and DG (b); Figure S7: The relative percentage (%) of normalized peak area abundances of the top five most abundant lipid molecular species of each lipid class identified in *Dunaliella salina* lipid extract (LE) using C18 RP-LC-MS; Table S1: Composition of the mix of phospholipid standards used in the analysis performed using C18 RP-LC-MS and MS/MS; Table S2: Lipid molecular species identified in the lipid extracts (LE) of *Dunaliella salina* by C18 RP-LC-MS and MS/MS, in positive and negative ion modes. Information on lipid species, chemical formulas, fatty acyl compositions, theoretical and observed masses, and mass errors (ppm) is shown. C:N/C:N means the attribution of fatty acyl chains to the position *sn*-1/*sn*-2, while C:N_C:N means that the attribution of the *sn*-position of fatty acyl chains is not known; Table S3: Lipid molecular species identified in lipid extracts of *Dunaliella salina* by C18-HPLC-ESI-MS and MS/MS. PC: phosphatidylcholine; LPC: lyso phosphatidylcholine; PG: phosphatidylglycerol; PI: phosphatidylinositol; MGDG: monogalactosyl diacylglycerol; MGMG: monogalatosyl monoacylglycerol; DGDG: digalactosyl diacylglycerol; DGMG: digalactosyl monoacylglycerol; SQDG: sulfoquinovosyl diacylglycerol; SQMG: sulfoquinovosyl monoacylglycerol; DGTS: diacylglyceryl-*N*,*N*,*N*-trimethyl homoserine; MGTS: monoacylglyceryl-*N*,*N*,*N*-trimethyl homoserine; Cer: ceramide; PI-Cer: inositolphosphoceramide; TG: triacylglycerols; DG: diacylglycerols. Manual curation was used to adjust the integration boundaries of chromatographic peaks as necessary; Table S4: Non-normalized datasets that represent the peak areas of all lipid molecular species identified in *Dunaliella salina* lipid extracts by the C18-HPLC-ESI-MS and MS/MS method reported in this study. PC: phosphatidylcholine; LPC: lyso phosphatidylcholine; PG: phosphatidylglycerol; PI: phosphatidylinositol; MGDG: monogalactosyl diacylglycerol; MGMG: monogalatosyl monoacylglycerol; DGDG: digalactosyl diacylglycerol; DGMG: digalactosyl monoacylglycerol; SQDG: sulfoquinovosyl diacylglycerol; SQMG: sulfoquinovosyl monoacylglycerol; DGTS: diacylglyceryl-*N*,*N*,*N*-trimethyl homoserine; MGTS: monoacylglyceryl-*N*,*N*,*N*-trimethyl homoserine; Cer: ceramide; PI-Cer: inositolphosphoceramide; TG: triacylglycerols; DG: diacylglycerols; Table S5: Normalized datasets (by internal standards) of all lipid molecular species identified *in Dunaliella salina* lipid extracts by the C18-HPLC-ESI-MS and MS/MS method reported in this study. PC: phosphatidylcholine; LPC: lyso phosphatidylcholine; PG: phosphatidylglycerol; PI: phosphatidylinositol; MGDG: monogalactosyl diacylglycerol; MGMG: monogalatosyl monoacylglycerol; DGDG: digalactosyl diacylglycerol; DGMG: digalactosyl monoacylglycerol; SQDG: sulfoquinovosyl diacylglycerol; SQMG: sulfoquinovosyl monoacylglycerol; DGTS: diacylglyceryl-*N*,*N*,*N*-trimethyl homoserine; MGTS: monoacylglyceryl-*N*,*N*,*N*-trimethyl homoserine; Cer: ceramide; PI-Cer: inositolphosphoceramide; TG: triacylglycerols; DG: diacylglycerols; Table S6: Normalized datasets (by the sum) of all lipid molecular species identified in *Dunaliella salina* lipid extracts by the C18-HPLC-ESI-MS and MS/MS method reported in this study. PC: phosphatidylcholine; LPC: lyso phosphatidylcholine; PG: phosphatidylglycerol; PI: phosphatidylinositol; MGDG: monogalactosyl diacylglycerol; MGMG: monogalatosyl monoacylglycerol; DGDG: digalactosyl diacylglycerol; DGMG: digalactosyl monoacylglycerol; SQDG: sulfoquinovosyl diacylglycerol; SQMG: sulfoquinovosyl monoacylglycerol; DGTS: diacylglyceryl-*N*,*N*,*N*-trimethyl homoserine; MGTS: monoacylglyceryl-*N*,*N*,*N*-trimethyl homoserine; Cer: ceramide; PI-Cer: inositolphosphoceramide; TG: triacylglycerols; DG: diacylglycerols; Table S7: Relative percentage (%) of the normalized peak areas of the lipid molecular species within each lipid class identified in *Dunaliella salina* lipid extracts by the C18-HPLC-ESI-MS and MS/MS method reported in this study. PC: phosphatidylcholine; LPC: lyso phosphatidylcholine; PG: phosphatidylglycerol; PI: phosphatidylinositol; MGDG: monogalactosyl diacylglycerol; MGMG: monogalatosyl monoacylglycerol; DGDG: digalactosyl diacylglycerol; DGMG: digalactosyl monoacylglycerol; SQDG: sulfoquinovosyl diacylglycerol; SQMG: sulfoquinovosyl monoacylglycerol; DGTS: diacylglyceryl-*N*,*N*,*N*-trimethyl homoserine; MGTS: monoacylglyceryl-*N*,*N*,*N*-trimethyl homoserine; Cer: ceramide; PI-Cer: inositolphosphoceramide; TG: triacylglycerols; DG: diacylglycerols.
