# Supplementary material for: Bioactive Lipids in Dunaliella salina: Implications for Functional Foods and Health
Source: Foods. 2024 Oct 19;13(20):3321. doi: 10.3390/foods13203321 (PMC11507028; doi:10.3390/foods13203321)
Supplement: Supplementary file 1 [file foods-13-03321-s001.zip › foods-3238506-supplementary_final.pdf]

Table S1: Composition of the mix of phospholipid standards used in the analysis performed using C18 RP-LC-MS and MS/MS.

Lipid standard	Formula	Fatty Acyl Chains	Theorical *m/z*	Quantity µg
dMPC	$C_{36}H_{73}NO_8P$	14:0/14:0	678.5074	0.04
LPC	$C_{27}H_{57}NO_7P$	19:0	538.3873	0.04
dMPG	$C_{34}H_{66}O_{10}P$	14:0/14:0	665.4394	0.024
dPPI	$C_{41}H_{78}O_{13}P$	16:0/16:0	809.5180	0.08
dMPE	$C_{33}H_{65}NO_8P$	14:0/14:0	634.4448	0.04
dMPS	$C_{34}H_{65}NO_{10}P$	14:0/14:0	678.4346	0.08
dMPA	$C_{31}H_{60}O_8P$	14:0/14:0	591.4026	0.16
CL 56:0	$C_{65}H_{125}O_{17}P_2$	14:0/14:0/14:0/14:0	1239.8392	0.16
SM 35:1;O2	$C_{41}H_{82}N_2O_8P$	18:1;O2/17:0	761.5809	0.04
Cer 35:1;O2	$C_{35}H_{70}NO_3$	18:1;O2/17:0	552.5356	0.08

Table S2: Lipid molecular species identified in the lipid extracts (LE) of *Dunaliella salina* by C18 RP-LC-MS and MS/MS, in positive and negative ion modes. Information on lipid species, chemical formulas, fatty acyl compositions, theoretical and observed masses, and mass errors (ppm) is shown. C:N/C:N means the attribution of fatty acyl chains to the position *sn*-1/*sn*-2, while C:N_C:N means that the attribution of the *sn*-position of fatty acyl chains is not known.

Lipid Species (C:N)	Formula	Fatty Acyl Chains	Theorical *m/z*	Observed *m/z*	Error (ppm)
		$[M+H]^+$			
PC O-31:0	$C_{39}H_{81}NO_7P$	-	706.5751	706.5732	-2.6423
PC 31:1	$C_{39}H_{77}NO_8P$	-	718.5387	718.5389	0.3034
PC 32:4_1	$C_{40}H_{73}NO_8P$	-	726.5074	726.5052	-3.0034
PC 32:4_2	$C_{40}H_{73}NO_8P$	-	726.5074	726.5049	-3.4163
PC 32:4_3	$C_{40}H_{73}NO_8P$	-	726.5074	726.5057	-2.3152
PC O-34:3_1 / P-34:2_1	$C_{42}H_{81}NO_7P$	-	742.5751	742.5729	-2.9182
PC O-34:3_2 / P-34:2_2	$C_{42}H_{81}NO_7P$	-	742.5751	742.5726	-3.3222
PC 33:2_1	$C_{41}H_{79}NO_8P$	-	744.5543	744.5521	-2.9964
PC 33:2_2	$C_{41}H_{79}NO_8P$	-	744.5543	744.5517	-3.5337
PC O-34:2_1	$C_{42}H_{83}NO_7P$	-	744.5907	744.5882	-3.3804
PC O-34:2_2	$C_{42}H_{83}NO_7P$	-	744.5907	744.5899	-1.0972
PC 34:3	$C_{42}H_{79}NO_8P$	16:0_18:3	756.5543	756.5511	-4.2297
PC 34:2	$C_{42}H_{81}NO_8P$	16:0_18:2; 16:1_18:1	758.5700	758.5666	-4.4584
PC 34:1	$C_{42}H_{83}NO_8P$	16:0_18:1	760.5856	760.5825	-4.1179
PC O-36:4	$C_{44}H_{83}NO_7P$	-	768.5907	768.5907	0.0000
PC O-36:1_1	$C_{44}H_{89}NO_7P$	-	774.6377	774.6365	-1.5065
PC O-36:1_2	$C_{44}H_{89}NO_7P$	-	774.6377	774.6374	-0.3447
PC 36:6_1	$C_{44}H_{77}NO_8P$	-	778.5387	778.5366	-2.6742
PC 36:6_2	$C_{44}H_{77}NO_8P$	-	778.5387	778.5359	-3.5734
PC 36:6_3	$C_{44}H_{77}NO_8P$	-	778.5387	778.5352	-4.4725
PC 36:5_1	$C_{44}H_{79}NO_8P$	-	780.5543	780.5517	-3.3707

PC 36:5_2	$C_{44}H_{79}NO_8P$	-	780.5543	780.5507	-4.6518
PC 36:4_1	$C_{44}H_{81}NO_8P$	-	782.5700	782.5680	-2.5327
PC 36:4_2	$C_{44}H_{81}NO_8P$	-	782.5700	782.5670	-3.8105
PC 36:4_3	$C_{44}H_{81}NO_8P$	-	782.5700	782.5686	-1.7660
PC 36:3_1	$C_{44}H_{83}NO_8P$	-	784.5856	784.5835	-2.7174
PC 36:3_2	$C_{44}H_{83}NO_8P$	-	784.5856	784.5821	-4.5017
PC 36:2	$C_{44}H_{85}NO_8P$	18:1/18:1	786.6013	786.5990	-2.9011
PC 37:2_1	$C_{45}H_{87}NO_8P$	-	800.6169	800.6142	-3.4124
PC 37:2_2	$C_{45}H_{87}NO_8P$	-	800.6169	800.6130	-4.9112
PC 36:4;O2	$C_{44}H_{81}NO_{10}P$	-	814.5598	814.5571	-3.3294
PC 38:2_1	$C_{46}H_{89}NO_8P$	-	814.6326	814.6346	2.4772
PC 38:2_2	$C_{46}H_{89}NO_8P$	-	814.6326	814.6298	-3.4150
		**$[M+H]^+$**			
LPC 16:1	$C_{24}H_{49}NO_7P$	-	494.3247	494.3242	-0.9447
LPC 16:0	$C_{24}H_{51}NO_7P$	-	496.3403	496.3399	-0.8381
LPC 18:3	$C_{26}H_{49}NO_7P$	-	518.3247	518.3240	-1.3505
LPC 18:2	$C_{26}H_{51}NO_7P$	-	520.3403	520.3395	-1.5375
		**$[M-H]^-$**			
PG 32:1_1	$C_{38}H_{72}O_{10}P$	16:0_16:1; 14:0_18:1	719.4863	719.4869	0.8339
PG 32:1_2	$C_{38}H_{72}O_{10}P$	-	719.4863	719.4865	0.2780
PG 32:0	$C_{38}H_{74}O_{10}P$	16:0/16:0	721.5020	721.5019	-0.1386
PG 33:1	$C_{39}H_{74}O_{10}P$	16:0_17:1	733.5020	733.5026	0.8698
PG 34:4	$C_{40}H_{70}O_{10}P$	16:1_18:3	741.4707	741.4714	0.9441
PG 34:3_1	$C_{40}H_{72}O_{10}P$	16:0_18:3	743.4863	743.4862	-0.1345
PG 34:3_2	$C_{40}H_{72}O_{10}P$	16:1_18:2	743.4863	743.4868	0.6725
PG 34:2_1	$C_{40}H_{74}O_{10}P$	16:1_18:1	745.5020	745.5026	0.8048
PG 34:2_2	$C_{40}H_{74}O_{10}P$	16:0_18:2	745.5020	745.5028	1.0731
PG 34:1	$C_{40}H_{76}O_{10}P$	16:0_18:1	747.5176	747.5186	1.3378
PG 34:4;O_1	$C_{40}H_{70}O_{11}P$	16:1_18:3;O	757.4656	757.4659	0.4251
PG 34:4;O_2	$C_{40}H_{70}O_{11}P$	-	757.4656	757.4650	-0.7631
PG 34:4;O_3	$C_{40}H_{70}O_{11}P$	-	757.4656	757.4647	-1.1591
PG 35:2	$C_{41}H_{76}O_{10}P$	16:1_19:1; 17:1_18:1	759.5176	759.5183	0.9045
PG 35:1	$C_{41}H_{78}O_{10}P$	16:0_19:1	761.5333	761.5333	0.0486
PG 34:4;O2_1	$C_{40}H_{70}O_{12}P$	16:1_18:3;O2	773.4605	773.4611	0.7848
PG 34:4;O2_2	$C_{40}H_{70}O_{12}P$	-	773.4605	773.4600	-0.6374
PG 36:2	$C_{42}H_{78}O_{10}P$	18:1/18:1	773.5333	773.5340	0.9049
PG 34:3;O2	$C_{40}H_{72}O_{12}P$	16:1_18:2;O2	775.4761	775.4763	0.2025
PG 37:2	$C_{43}H_{80}O_{10}P$	18:1_19:1	787.5489	787.5494	0.6184
		**$[M-H]^-$**			
PI 34:3	$C_{43}H_{76}O_{13}P$	16:0_18:3	831.5024	831.5031	0.8418
PI 34:2	$C_{43}H_{78}O_{13}P$	16:0_18:2	833.5180	833.5188	0.9598
PI 34:1	$C_{43}H_{80}O_{13}P$	16:0_18:1	835.5337	835.5346	1.0772
		**$[M+NH_4]^+$**			
MGDG 34:7	$C_{43}H_{72}NO_{10}$	16:4_18:3	762.5156	762.5141	-1.9986
MGDG 34:6_1	$C_{43}H_{74}NO_{10}$	16:3_18:3	764.5313	764.5294	-2.4499

MGDG 34:6_2	$C_{43}H_{74}NO_{10}$	16:4_18:2	764.5313	764.5293	-2.5807
MGDG 34:5_1	$C_{43}H_{76}NO_{10}$	16:2_18:3	766.5469	766.5452	-2.2490
MGDG 34:5_2	$C_{43}H_{76}NO_{10}$	16:4_18:1	766.5469	766.5450	-2.5100
MGDG 34:5_3	$C_{43}H_{76}NO_{10}$	-	766.5469	766.5489	2.5778
MGDG 34:4_1	$C_{43}H_{78}NO_{10}$	16:2_18:2; 16:1_18:3	768.5620	768.5603	-2.2119
MGDG 34:4_2	$C_{43}H_{78}NO_{10}$	-	768.5620	768.5591	-3.7733
MGDG 34:3_1	$C_{43}H_{80}NO_{10}$	-	770.5782	770.5754	-3.6648
MGDG 34:3_2	$C_{43}H_{80}NO_{10}$	16:1_18:2; 16:2_18:1	770.5782	770.5755	-3.5350
MGDG 34:2	$C_{43}H_{82}NO_{10}$	16:1_18:1; 16:0_18:2	772.5933	772.5921	-1.5532
MGDG 34:1_1	$C_{43}H_{84}NO_{10}$	-	774.6090	774.6074	-2.0656
MGDG 34:1_2	$C_{43}H_{84}NO_{10}$	16:0_18:1	774.6090	774.6077	-1.6783
		**$[M+NH_4]^+$**			
MGMG 16:0	$C_{25}H_{52}NO_9$	16:0	510.3642	510.3625	-3.3310
MGMG 18:3	$C_{27}H_{50}NO_9$	18:3	532.3486	532.3470	-2.9285
		**$[M+NH_4]^+$**			
DGDG 30:0	$C_{45}H_{88}O_{15}N$	14:0_16:0	882.6154	882.6118	-4.0788
DGDG 32:3	$C_{47}H_{86}O_{15}N$	14:0_18:3	904.5997	904.5973	-2.6531
DGDG 32:2	$C_{47}H_{88}O_{15}N$	14:0_18:2	906.6154	906.6126	-3.0884
DGDG 32:1	$C_{47}H_{90}O_{15}N$	14:0_18:1	908.6310	908.6278	-3.5218
DGDG 32:0	$C_{47}H_{92}O_{15}N$	16:0/16:0	910.6467	910.6432	-3.8434
DGDG 34:7	$C_{49}H_{82}O_{15}N$	16:4_18:3	924.5684	924.5656	-3.0284
DGDG 34:6	$C_{49}H_{84}O_{15}N$	16:3_18:3	926.5841	926.5812	-3.1298
DGDG 34:5	$C_{49}H_{86}O_{15}N$	16:2_18:3	928.5997	928.5967	-3.2307
DGDG 34:4_1	$C_{49}H_{88}O_{15}N$	-	930.6154	930.6122	-3.4386
DGDG 34:4_2	$C_{49}H_{88}O_{15}N$	16:1_18:3	930.6154	930.6128	-2.7939
DGDG 34:3	$C_{49}H_{90}O_{15}N$	16:1_18:2; 16:2_18:1	932.6310	932.6284	-2.7878
DGDG 34:2	$C_{49}H_{92}O_{15}N$	16:1_18:1; 16:0_18:2	934.6467	934.6427	-4.2797
DGDG 34:1	$C_{49}H_{94}O_{15}N$	16:0_18:1	936.6623	936.6594	-3.0961
DGDG 34:0	$C_{49}H_{96}O_{15}N$	16:0_18:0	938.6780	938.6759	-2.2372
DGDG 34:4;O	$C_{49}H_{88}O_{16}N$	16:0_18:4;O	946.6103	946.6074	-3.0784
DGDG 34:3;O_1	$C_{49}H_{90}O_{16}N$	-	948.6260	948.6225	-3.6516
DGDG 34:3;O_2	$C_{49}H_{90}O_{16}N$	16:0_18:3;O	948.6260	948.6236	-2.4920
DGDG 34:2;O_1	$C_{49}H_{92}O_{16}N$	-	950.6416	950.6381	-3.6965
DGDG 34:2;O_2	$C_{49}H_{92}O_{16}N$	16:0_18:2;O	950.6416	950.6404	-1.2770
DGDG 34:1;O_1	$C_{49}H_{94}O_{16}N$	-	952.6573	952.6563	-1.0109
DGDG 34:1;O_2	$C_{49}H_{94}O_{16}N$	16:0_18:1;O	952.6573	952.6540	-3.4252
DGDG 36:6	$C_{51}H_{88}O_{15}N$	18:3/18:3; 18:2_18:4	954.6154	954.6125	-3.0379
DGDG 36:5	$C_{51}H_{90}O_{15}N$	18:2_18:3	956.6310	956.6284	-2.7179
DGDG 36:4	$C_{51}H_{92}O_{15}N$	18:1_18:3; 18:2/18:2	958.6467	958.6440	-2.8165
DGDG 34:5;O2	$C_{49}H_{86}O_{17}N$	16:0_18:5;2O; 16:2_18:3;2O	960.5896	960.5860	-3.7258
DGDG 36:3_1	$C_{51}H_{94}O_{15}N$	-	960.6623	960.6580	-4.4761

DGDG 36:3_2	$C_{51}H_{94}O_{15}N$	18:1_18:2	960.6623	960.6625	0.2082
DGDG 34:4;O2_1	$C_{49}H_{88}O_{17}N$	-	962.6052	962.6010	-4.3933
DGDG 34:4;O2_2	$C_{49}H_{88}O_{17}N$	-	962.6052	962.6012	-4.1855
DGDG 36:2_1	$C_{51}H_{96}O_{15}N$	-	962.6780	962.6758	-2.2853
DGDG36:2_2	$C_{51}H_{96}O_{15}N$	18:1/18:1	962.6780	962.6757	-2.3892
DGDG 34:3;O2	$C_{49}H_{90}O_{17}N$	16:0_18:3;O2	964.6209	964.6177	-3.2956
DGDG 34:4;O3	$C_{49}H_{88}O_{18}N$	16:0_18:4;O3	978.6001	978.5961	-4.1324
		**$[M+NH_4]^+$**			
DGMG 16:0	$C_{31}H_{62}NO_{14}$	16:0	672.4170	672.4171	0.1487
DGMG 18:3	$C_{33}H_{60}NO_{14}$	18:3	694.4014	694.4005	-1.2730
		**$[M-H]^-$**			
SQDG 30:0_1	$C_{39}H_{73}O_{12}S$	-	765.4823	765.4789	-4.4116
SQDG 30:0_2	$C_{39}H_{73}O_{12}S$	-	765.4823	765.4791	-4.1503
SQDG 30:0_3	$C_{39}H_{73}O_{12}S$	-	765.4823	765.4827	0.5526
SQDG 32:0	$C_{41}H_{77}O_{12}S$	-	793.5136	793.5133	-0.3478
SQDG 34:3	$C_{43}H_{75}O_{12}S$	-	815.4979	815.4981	0.2121
SQDG 34:2	$C_{43}H_{77}O_{12}S$	-	817.5136	817.5141	0.6410
SQDG 34:1	$C_{43}H_{79}O_{12}S$	-	819.5292	819.5293	0.0891
SQDG 34:0	$C_{43}H_{81}O_{12}S$	-	821.5449	821.5462	1.6104
SQDG 34:4;O	$C_{43}H_{73}O_{13}S$	-	829.4772	829.4775	0.3713
SQDG 34:3;O_1	$C_{43}H_{75}O_{13}S$	-	831.4928	831.4937	1.0319
SQDG 34:3;O_2	$C_{43}H_{75}O_{13}S$	-	831.4928	831.4935	0.7913
SQDG 35:1	$C_{44}H_{81}O_{12}S$	-	833.5449	833.5459	1.2273
SQDG 36:2	$C_{45}H_{81}O_{12}S$	-	845.5449	845.5444	-0.5641
SQDG 36:1	$C_{45}H_{83}O_{12}S$	-	847.5605	847.5612	0.7940
SQDG 34:3;O3	$C_{43}H_{75}O_{15}S$	-	863.4827	863.4837	1.1905
SQDG 34:3;O4	$C_{43}H_{75}O_{16}S$	-	879.4776	879.4783	0.8107
SQMG 16:0	$C_{25}H_{47}O_{11}S$	-	555.2839	555.2850	1.9594
		**$[M+H]^+$**			
DGTS 32:1	$C_{42}H_{80}O_7N$	14:0_18:1; 16:0_16:1	710.5935	710.5913	-3.0960
DGTS 32:0	$C_{42}H_{82}O_7N$	16:0/16:0	712.6091	712.6075	-2.2453
DGTS 33:3_1	$C_{43}H_{78}O_7N$	15:0_18:3; 16:0_17:3	720.5778	720.5752	-3.6082
DGTS 33:3_2	$C_{43}H_{78}O_7N$	-	720.5778	720.5749	-4.0245
DGTS 33:2	$C_{43}H_{80}O_7N$	15:0_18:2; 16:0_17:2; 16:1_17:1	722.5935	722.5918	-2.3236
DGTS 34:6_1	$C_{44}H_{74}O_7N$	16:4_18:2	728.5465	728.5448	-2.3334
DGTS 34:6_2	$C_{44}H_{74}O_7N$	-	728.5465	728.5433	-4.3923
DGTS 34:5_1	$C_{44}H_{76}O_7N$	16:2_18:3; 16:3_18:2; 16:4_18:1	730.5622	730.5603	-2.6007
DGTS 34:5_2	$C_{44}H_{76}O_7N$	-	730.5622	730.5594	-3.8327
DGTS 34:4_1	$C_{44}H_{78}O_7N$	16:1_18:3; 16:2_18:2; 16:0_18:4	732.5778	732.5750	-3.8221
DGTS 34:4_2	$C_{44}H_{78}O_7N$	-	732.5778	732.5740	-5.1872

DGTS 34:3	$C_{44}H_{80}O_7N$	16:1_18:2; 16:0_18:3; 16:2_18:1	734.5935	734.5912	-3.1310
DGTS 34:2	$C_{44}H_{82}O_7N$	16:0_18:2	736.6091	736.6070	-2.8509
DGTS 34:1	$C_{44}H_{84}O_7N$	16:0_18:1	738.6248	738.6220	-3.7908
DGTS 34:4;O	$C_{44}H_{78}O_8N$	16:0_18:4;O	748.5727	748.5701	-3.5321
DGTS 35:3_1	$C_{45}H_{82}O_7N$	-	748.6091	748.6065	-3.5105
DGTS 35:3_2	$C_{45}H_{82}O_7N$	17:0_18:3	748.6091	748.6068	-3.1098
DGTS 36:6_1	$C_{46}H_{78}O_7N$	18:3/18:3	756.5778	756.5754	-3.1722
DGTS 36:6_2	$C_{46}H_{78}O_7N$	-	756.5778	756.5742	-4.7583
DGTS 36:5_1	$C_{46}H_{80}O_7N$	18:2_18:3	758.5935	758.5908	-3.5592
DGTS 36:5_2	$C_{46}H_{80}O_7N$	-	758.5935	758.5900	-4.6138
DGTS 36:4_1	$C_{46}H_{82}O_7N$	18:2/18:2; 18:1_18:3	760.6091	760.6062	-3.8127
DGTS 36:4_2	$C_{46}H_{82}O_7N$	18:1_18:3	760.6091	760.6059	-4.2072
DGTS 34:5;O2	$C_{44}H_{76}O_9N$	-	762.5520	762.5503	-2.2412
DGTS 36:3_1	$C_{46}H_{84}O_7N$	-	762.6248	762.6224	-3.1470
DGTS 36:3_2	$C_{46}H_{84}O_7N$	18:1_18:2	762.6248	762.6216	-4.1960
DGTS 34:4;O2	$C_{44}H_{78}O_9N$	16:0_18:4;O2	764.5677	764.5653	-3.0854
DGTS 36:2	$C_{46}H_{86}O_7N$	18:1/18:1	764.6404	764.6383	-2.7464
DGTS 34:3;O2	$C_{44}H_{80}O_9N$	16:0_18:3;O2	766.5833	766.5811	-2.8816
DGTS 36:1	$C_{46}H_{88}O_7N$	18:0_18:1	766.6561	766.6535	-3.3914
DGTS 36:6;O_1	$C_{46}H_{78}O_8N$	18:2;O_18:4; 18:3_18:3;O	772.5727	772.5708	-2.5163
DGTS 36:6;O_2	$C_{46}H_{78}O_8N$	-	772.5727	772.5695	-4.1990
DGTS 36:5;O_1	$C_{46}H_{80}O_8N$	18:2;O_18:3	774.5884	774.5869	-1.9288
DGTS 36:5;O_2	$C_{46}H_{80}O_8N$	-	774.5884	774.5851	-4.2526
DGTS 37:4	$C_{47}H_{84}O_7N$	18:3_19:1	774.6248	774.6223	-3.2003
DGTS 36:4;O_1	$C_{46}H_{82}O_8N$	18:1;O_18:3; 18:2_18:2;O	776.6040	776.6011	-3.7896
DGTS 36:4;O_2	$C_{46}H_{82}O_8N$	18:1_18:3;O	776.6040	776.6010	-3.9183
DGTS 37:3_1	$C_{47}H_{86}O_7N$	18:2_19:1	776.6404	776.6374	-3.9001
DGTS 37:3_2	$C_{47}H_{86}O_7N$	-	776.6404	776.6438	4.3405
DGTS 36:3;O_1	$C_{46}H_{84}O_8N$	18:0;O_18:3; 18:1_18:2;O	778.6197	778.6161	-4.6159
DGTS 36:3;O_2	$C_{46}H_{84}O_8N$	16:0_20:3;O	778.6197	778.6170	-3.4600
DGTS 37:2	$C_{47}H_{88}O_7N$	18:1_19:1	778.6561	778.6532	-3.6974
		**$[M+H]^+$**			
MGTS 16:1	$C_{26}H_{50}O_6N$	16:1	472.3638	472.3624	-2.9935
MGTS 16:0	$C_{26}H_{52}O_6N$	16:0	474.3795	474.3786	-1.8972
MGTS 18:3	$C_{28}H_{50}O_6N$	18:3	496.3638	496.3629	-1.8414
MGTS 18:2	$C_{28}H_{52}O_6N$	18:2	498.3795	498.3782	-2.5362
MGTS 18:1	$C_{28}H_{54}O_6N$	18:1	500.3951	500.3937	-2.8258
MGTS 18:3;O2	$C_{28}H_{50}O_8N$	18:3;O2	528.3536	528.3545	1.6201
MGTS 18:2;O2	$C_{28}H_{52}O_8N$	18:2;O2	530.3693	530.3701	1.5197
MGTS 18:3;O3	$C_{28}H_{50}O_9N$	18:3;O3	544.3486	544.3491	0.9938
MGTS 18:2;O3	$C_{28}H_{52}O_9N$	18:2;O3	546.3642	546.3650	1.4478
		**$[M+H]^+$**			

Cer 34:2;O_1	$C_{34}H_{66}NO_2$	-	520.5094	520.5080	-2.6013
Cer 34:2;O_2	$C_{34}H_{66}NO_2$	18:2;O/16:0; 17:1;O/17:1	520.5094	520.5086	-1.4486
Cer 33:0;O2_1	$C_{33}H_{68}NO_3$	-	526.5199	526.5184	-2.8850
Cer 33:0;O2_2	$C_{33}H_{68}NO_3$	17:0;O2/16:0	526.5199	526.5189	-1.9353
Cer 34:3;O2_1	$C_{34}H_{64}NO_3$	-	534.4886	534.4877	-1.7194
Cer 34:3;O2_2	$C_{34}H_{64}NO_3$	19:1;O2/15:2; 19:2;O2/15:1	534.4886	534.4879	-1.3452
Cer 35:2;O	$C_{35}H_{68}NO_2$	18:2;O/17:0; 17:1;O/18:1	534.5250	534.5242	-1.5041
Cer 34:1;O2_1	$C_{34}H_{68}NO_3$	18:1;O2/16:0	538.5199	538.5193	-1.1494
Cer 34:1;O2_2	$C_{34}H_{68}NO_3$	-	538.5199	538.5190	-1.7065
Cer 34:0;O2_1	$C_{34}H_{70}NO_3$	18:0;O2/16:0	540.5356	540.5342	-2.5327
Cer 34:0;O2_2	$C_{34}H_{70}NO_3$	-	540.5356	540.5349	-1.2377
Cer 34:0;O2_3	$C_{34}H_{70}NO_3$	-	540.5356	540.5343	-2.3477
Cer 34:0;O2_4	$C_{34}H_{70}NO_3$	-	540.5356	540.5336	-3.6427
Cer 35:0;O2_1	$C_{35}H_{72}NO_3$	19:0;O2/16:0; 17:0;O2/18:0	554.5512	554.5506	-1.1162
Cer 35:0;O2_2	$C_{35}H_{72}NO_3$	18:0;O2/17:0	554.5512	554.5507	-0.9359
Cer 35:0;O2_3	$C_{35}H_{72}NO_3$	-	554.5512	554.5501	-2.0178
Cer 37:1;O2	$C_{37}H_{74}NO_3$	18:1;O2/19:0; 17:0;O2/20:1	580.5669	580.5654	-2.5303
Cer 37:1;O3_1	$C_{37}H_{74}NO_4$	-	596.5618	596.5615	-0.4761
Cer 37:1;O3_2	$C_{37}H_{74}NO_4$	18:0;O3/19:1	596.5618	596.5615	-0.4761
Cer 37:1;O3_3	$C_{37}H_{74}NO_4$	-	596.5618	596.5605	-2.1523
Cer 48:0;O3	$C_{48}H_{98}NO_4$	22:0;O3/26:0	752.7496	752.7464	-4.2298
PI-Cer 34:1;O2	$C_{40}H_{77}NO_{11}P$	20:1;O2/14:0	778.5234	778.5241	0.8645
		**$[M+NH_4]^+$**			
TG 40:0	$C_{43}H_{86}O_6N$	8:0_16:0_16:0; 10:0_14:0_16:0	712.6455	712.6431	-3.3874
TG 44:0	$C_{47}H_{94}O_6N$	13:0_15:0_16:0; 12:0_16:0_16:0; 13:0_14:0_17:0	768.7081	768.7053	-3.6607
TG 45:1_1	$C_{48}H_{94}O_6N$	-	780.7081	780.7050	-3.9887
TG 45:1_2	$C_{48}H_{94}O_6N$	-	780.7081	780.7056	-3.2202
TG 45:1_3	$C_{48}H_{94}O_6N$	13:1_16:0_16:0; 14:0_15:0_16:1; 14:0_15:1_16:0; 13:1_14:0_18:0	780.7081	780.7052	-3.7325
TG 45:0_1	$C_{48}H_{96}O_6N$	-	782.7238	782.7210	-3.5313
TG 45:0_2	$C_{48}H_{96}O_6N$	13:0_16:0_16:0; 14:0_15:0_16:0; 14:0_14:0_17:0	782.7238	782.7206	-4.0423
TG 46:4_1	$C_{49}H_{90}O_6N$	-	788.6768	788.6758	-1.2857
TG 46:4_2	$C_{49}H_{90}O_6N$	14:0_16:0_16:4	788.6768	788.6750	-2.3001

TG 46:1	$C_{49}H_{96}O_6N$	12:0_16:0_18:1; 14:0_16:0_16:1; 14:1_16:0_16:0	794.7238	794.7207	-3.8554
TG 46:0	$C_{49}H_{98}O_6N$	14:0_16:0_16:0	796.7394	796.7356	-4.7870
TG 47:2_1	$C_{50}H_{96}O_6N$	-	806.7238	806.7208	-3.6741
TG 47:2_2	$C_{50}H_{96}O_6N$	13:0_16:0_18:2; 13:0_16:1_18:1; 14:0_15:0_18:2; 14:0_15:1_18:1; 14:0_16:1_17:1; 15:0_15:1_17:1; 15:0_16:1_16:1	806.7238	806.7205	-4.0460
TG 47:1_1	$C_{50}H_{98}O_6N$		808.7394	808.7373	-2.6139
TG 47:1_2	$C_{50}H_{98}O_6N$	15:0_16:0_16:1;	808.7394	808.7360	-4.2214
TG 47:0_1	$C_{50}H_{100}O_6N$	-	810.7551	810.7519	-3.9025
TG 47:0_2	$C_{50}H_{100}O_6N$	14:0_15:0_18:0; 14:0_16:0_17:0; 15:0_15:0_17:0; 15:0_16:0_16:0	810.7551	810.7517	-4.1492
TG 48:5	$C_{51}H_{92}O_6N$	14:0_16:2_18:3; 14:0_16:3_18:2	814.6925	814.6902	-2.7790
TG 48:4	$C_{51}H_{94}O_6N$	12:0_18:1_18:3; 12:0_18:2_18:2; 14:0_16:1_18:3; 14:0_16:2_18:2; 16:1_16:1_16:2	816.7081	816.7051	-3.6904
TG 48:3	$C_{51}H_{96}O_6N$	14:0_16:0_18:3; 14:0_16:1_18:2; 14:0_16:2_18:1; 14:1_16:0_18:2; 14:1_16:1_18:1; 14:2_16:0_18:1; 16:0_16:0_16:3; 16:0_16:1_16:2; 16:1_16:1_16:1	818.7238	818.7205	-3.9867
TG 48:2	$C_{51}H_{98}O_6N$	14:0_16:0_18:2; 14:0_16:1_18:1; 14:1_16:0_18:1; 14:1_16:1_18:0; 14:2_16:0_18:0; 16:0_16:0_16:2; 16:0_16:1_16:1	820.7394	820.7360	-4.1597
TG 48:0	$C_{51}H_{102}O_6N$	14:0_17:0_17:0; 15:0_16:0_17:0; 15:0_15:0_18:0; 14:0_16:0_18:0; 16:0_16:0_16:0	824.7707	824.7682	-3.0481

TG 49:3	$C_{52}H_{98}O_6N$	15:0_16:0_18:3; 15:1_16:0_18:2; 15:1_16:1_18:1; 15:1_17:1_17:1	832.7394	832.7356	-4.5801
TG 49:2_1	$C_{52}H_{100}O_6N$	-	834.7551	834.7525	-3.0716
TG 49:2_2	$C_{52}H_{100}O_6N$	13:0_18:1_18:2; 14:0_17:0_18:2; 14:0_17:1_18:1; 15:0_16:0_18:2; 15:0_16:1_18:1; 15:1_16:0_18:1; 16:0_16:1_17:1; 16:1_16:1_17:0	834.7551	834.7517	-4.0299
TG 49:1	$C_{52}H_{102}O_6N$	14:0_16:0_19:1; 14:0_17:0_18:1; 14:0_17:1_18:0; 15:0_16:0_18:1; 15:0_16:1_18:0; 15:0_17:0_17:1; 16:0_16:0_17:1; 16:0_16:1_17:0	836.7707	836.7672	-4.1995
TG 49:0_1	$C_{52}H_{104}O_6N$	-	838.7864	838.7826	-4.4874
TG 49:0_2	$C_{52}H_{104}O_6N$	14:0_16:0_19:0; 14:0_17:0_18:0; 15:0_16:0_18:0; 15:0_17:0_17:0; 16:0_16:0_17:0	838.7864	838.7827	-4.3682
TG 50:6	$C_{53}H_{94}O_6N$	16:0_16:4_18:2	840.7081	840.7052	-3.4661
TG 50:5	$C_{53}H_{96}O_6N$	14:0_18:2_18:3; 16:1_16:1_18:3; 16:1_16:2_18:2	842.7238	842.7206	-3.7545
TG 50:4	$C_{53}H_{98}O_6N$	14:2_18:1_18:1; 16:0_16:1_18:3; 16:0_16:2_18:2; 16:0_16:3_18:1	844.7394	844.7361	-3.9231
TG 50:3	$C_{53}H_{100}O_6N$	16:0_16:0_18:3	846.7551	846.7521	-3.5004
TG 50:2	$C_{53}H_{102}O_6N$	16:0_16:0_18:2	848.7707	848.7673	-4.0223
TG 50:1	$C_{53}H_{104}O_6N$	16:0_16:0_18:1	850.7864	850.7838	-3.0137
TG 50:0	$C_{53}H_{106}O_6N$	16:0_16:0_18:0	852.8020	852.7979	-4.8241
TG 51:3_1	$C_{54}H_{102}O_6N$	-	860.7707	860.7767	6.9542
TG 51:3_2	$C_{54}H_{102}O_6N$	16:0_17:0_18:3; 16:0_17:1_18:2; 16:1_17:1_18:1; 16:1_16:1_19:1	860.7707	860.7767	6.9542
TG 51:1	$C_{54}H_{106}O_6N$	15:0_17:0_19:1; 16:0_17:0_18:1	864.8020	864.7980	-4.6415
TG 51:0_1	$C_{54}H_{108}O_6N$	-	866.8177	866.8133	-5.0345

TG 51:0_2	$C_{54}H_{108}O_6N$	15:0_16:0_20:0; 15:0_17:0_19:0; 15:0_18:0_18:0; 16:0_16:0_19:0; 17:0_17:0_17:0	866.8177	866.8142	-3.9962
TG 52:6	$C_{55}H_{98}O_6N$	16:1_18:2_18:3; 16:2_18:1_18:3	868.7394	868.7363	-3.5845
TG 52:5	$C_{55}H_{100}O_6N$	16:1_18:1_18:3; 16:2_18:1_18:2	870.7551	870.7515	-4.0930
TG 52:4	$C_{55}H_{102}O_6N$	16:1_18:1_18:2; 16:2_18:1_18:1	872.7707	872.7669	-4.3700
TG 52:3	$C_{55}H_{104}O_6N$	16:0_18:0_18:3; 16:0_18:1_18:2; 16:1_18:0_18:2; 16:1_18:1_18:1	874.7864	874.7825	-4.4171
TG 52:2	$C_{55}H_{106}O_6N$	16:0_18:1_18:1; 14:2_16:0_22:0; 16:0_17:1_19:1; 16:0_18:0_18:2; 16:1_18:0_18:1	876.8020	876.7980	-4.5780
TG 52:0	$C_{55}H_{110}O_6N$	14:0_16:0_22:0; 14:0_18:0_20:0; 16:0_16:0_20:0; 16:0_18:0_18:0	880.8333	880.8296	-4.2165
TG 53:4	$C_{56}H_{104}O_6N$	16:0_18:3_19:1; 17:0_18:1_18:3; 17:0_18:2_18:2; 17:1_18:0_18:3; 17:2_18:0_18:2	886.7864	886.7829	-3.9062
TG 53:3	$C_{56}H_{106}O_6N$	16:1_17:1_20:1; 16:0_18:1_19:2; 16:0_18:2_19:1; 16:1_18:0_19:2; 16:1_18:1_19:1; 17:0_18:1_18:2; 17:1_18:0_18:2; 17:1_18:1_18:1	888.8020	888.7982	-4.2912
TG 53:1_1	$C_{56}H_{110}O_6N$	-	892.8333	892.8289	-4.9438
TG 53:1_2	$C_{56}H_{110}O_6N$	15:0_18:1_20:0; 15:0_19:0_19:1; 16:0_18:0_19:1; 16:0_18:1_19:0; 17:0_18:0_18:1	892.8333	892.8302	-3.4878
TG 53:0_1	$C_{56}H_{112}O_6N$	-	894.8490	894.8469	-2.3065

TG 53:0_2	$C_{56}H_{112}O_6N$	14:0_15:0_24:0; 14:0_17:0_22:0; 14:0_18:0_21:0; 16:0_15:0_22:0; 16:0_16:0_21:0; 16:0_17:0_20:0; 17:0_17:0_18:0	894.8490	894.8467	-2.5300
TG 54:6	$C_{57}H_{102}O_6N$	16:3_18:3_20:0; 18:0_18:2_18:4; 18:0_18:3_18:3; 18:1_18:2_18:3; 18:2_18:2_18:2	896.7707	896.7668	-4.3645
TG 54:5	$C_{57}H_{104}O_6N$	16:4_18:1_20:0; 18:1_18:2_18:2	898.7864	898.7821	-4.7442
TG 54:4	$C_{57}H_{106}O_6N$	18:1_18:1_18:2	900.8020	900.7978	-4.6781
TG 54:3	$C_{57}H_{108}O_6N$	16:0_18:3_20:0; 16:0_18:2_20:1; 16:2_18:0_20:1; 16:2_18:1_20:0; 18:0_18:0_18:3; 18:0_18:1_18:2; 18:1_18:1_18:1	902.8177	902.8135	-4.6122
TG 54:1	$C_{57}H_{112}O_6N$	14:0_18:1_22:0; 14:0_20:0_20:1; 16:0_18:0_20:0; 16:0_18:1_20:0; 18:0_18:0_18:1	906.8490	906.8449	-4.4815
TG 55:4	$C_{58}H_{108}O_6N$	18:1_18:1_19:2; 18:1_18:2_19:1	914.8177	914.8132	-4.8797
TG 55:3_1	$C_{58}H_{110}O_6N$	-	916.8333	916.8288	-4.9235
TG 55:3_2	$C_{58}H_{110}O_6N$	18:1_18:1_19:1	916.8333	916.8293	-4.3781
TG 55:2_1	$C_{58}H_{11}2O_6N$	-	918.8490	918.8446	-4.7494
TG 55:2_2	$C_{58}H_{112}O_6N$	16:0_18:1_21:1; 16:0_18:2_21:0; 16:1_18:1_21:0; 16:0_19:1_20:1; 16:1_19:0_20:1; 16:1_19:1_20:0; 17:1_18:0_20:1; 17:1_18:1_20:0; 17:1_19:0_19:1	918.8490	918.8463	-2.8993
TG 55:0_1	$C_{58}H_{116}O_6N$	-	922.8803	922.8762	-4.4036
TG 55:0_2	$C_{58}H_{116}O_6N$	14:0_16:0_25:0; 15:0_16:0_24:0; 16:0_16:0_23:0	922.8803	922.8769	-3.6451
TG 56:6_1	$C_{59}H_{106}O_6N$	-	924.8020	924.7979	-4.4485
TG 56:6_2	$C_{59}H_{106}O_6N$	20:0_18:3_18:3	924.8020	924.7978	-4.5567
TG 56:6_3	$C_{59}H_{106}O_6N$	-	924.8020	924.8029	0.9580
TG 56:5_1	$C_{59}H_{108}O_6N$	-	926.8177	926.8148	-3.0901

TG 56:5_2	$C_{59}H_{108}O_6N$	16:4_18:1_22:0	926.8177	926.8133	-4.7086
TG 56:5_3	$C_{59}H_{108}O_6N$	-	926.8177	926.8189	1.3336
TG 56:4_1	$C_{59}H_{110}O_6N$	-	928.8333	928.8301	-3.4603
TG 56:4_2	$C_{59}H_{110}O_6N$	18:1_18:3_20:0	928.8333	928.8291	-4.5369
TG 56:1	$C_{59}H_{116}O_6N$	16:0_22:0_18:1	934.8803	934.8769	-3.5983
TG 57:4_1	$C_{60}H_{112}O_6N$	-	942.8490	942.8446	-4.6285
TG 57:4_2	$C_{60}H_{112}O_6N$	-	942.8490	942.8449	-4.3103
TG 57:4_3	$C_{60}H_{112}O_6N$	18:1_18:3_21:0	942.8490	942.8459	-3.2497
TG 57:2_1	$C_{60}H_{116}O_6N$	-	946.8803	946.8778	-2.6022
TG 57:2_2	$C_{60}H_{116}O_6N$	16:1_19:1_22:0; 17:1_18:1_22:0; 17:1_19:1_21:0; 18:1_18:1_21:0; 18:1_19:1_20:0	946.8803	946.8774	-3.0247
TG 57:0_1	$C_{60}H_{120}O_6N$	-	950.9116	950.9080	-3.7480
TG 57:0_2	$C_{60}H_{120}O_6N$	15:0_16:0_26:0; 15:0_17:0_25:0; 15:0_18:0_24:0; 16:0_16:0_25:0; 16:0_17:0_24:0	950.9116	950.9096	-2.0654
TG 58:2	$C_{61}H_{118}O_6N$	16:0_16:1_26:1; 16:0_18:1_24:1; 16:0_20:1_22:1; 16:1_20:0_22:1; 16:1_20:1_22:0; 18:1_18:1_22:0; 18:1_20:0_20:1	960.8959	960.8919	-4.1774
TG 58:1_1	$C_{61}H_{120}O_6N$	-	962.9116	962.9078	-3.9090
TG 58:1_2	$C_{61}H_{120}O_6N$	16:0_18:1_24:0	962.9116	962.9083	-3.3897
TG 59:1_1	$C_{62}H_{122}O_6N$	-	976.9272	976.9226	-4.7230
TG 59:1_2	$C_{62}H_{122}O_6N$	16:0_18:1_25:0	976.9272	976.9239	-3.3923
TG 60:5	$C_{63}H_{116}O_6N$	16:4_18:1_26:0	982.8803	982.8753	-5.0000
TG 60:3_1	$C_{63}H_{120}O_6N$	-	986.9116	986.9083	-3.3073
TG 60:3_2	$C_{63}H_{120}O_6N$	16:0_18:3_26:0; 16:2_18:1_26:0	986.9116	986.9078	-3.8139
TG 60:1	$C_{63}H_{124}O_6N$	16:0_18:1_26:0	990.9429	990.9398	-3.0920
TG 61:2_1	$C_{64}H_{124}O_6N$	-	1002.9429	1002.9378	-5.0491
TG 61:2_2	$C_{64}H_{124}O_6N$	17:1_17:1_27:0; 17:1_18:1_26:0; 18:1_18:1_25:0	1002.9429	1002.9395	-3.3541
TG 62:5	$C_{65}H_{120}O_6N$	18:2_18:3_26:0	1010.9116	1010.9078	-3.7234
TG 62:1	$C_{65}H_{128}O_6N$	16:0_18:1_28:0; 18:0_18:1_26:0	1018.9742	1018.9707	-3.3995
TG 63:2_1	$C_{66}H_{128}O_6N$	-	1030.9742	1030.9709	-3.1659
TG 63:2_2	$C_{66}H_{128}O_6N$	18:1_18:1_27:0	1030.9742	1030.9714	-2.6810
TG 64:4	$C_{67}H_{126}O_6N$	18:1_18:3_28:0; 18:2_18:2_28:0	1040.9585	1040.9548	-3.5679

TG 64:2	$C_{67}H_{130}O_6N$	18:1_18:1_28:0; 18:1_20:1_26:0	1044.9898	1044.9862	-3.4584
		$[M+NH_4]^+$			
DG 34:3_1	$C_{37}H_{70}O_5N$	16:0_18:3	608.5254	608.5242	-1.9703
DG 34:3_2	$C_{37}H_{70}O_5N$	-	608.5254	608.5258	0.6590
DG 34:3_3	$C_{37}H_{70}O_5N$	16:0_18:3	608.5254	608.5253	-0.1627
DG 34:2_1	$C_{37}H_{72}O_5N$	-	610.5410	610.5394	-2.6992
DG 34:2_2	$C_{37}H_{72}O_5N$	16:0_18:2	610.5410	610.5409	-0.2424
DG 34:2_3	$C_{37}H_{72}O_5N$	-	610.5410	610.5401	-1.5527
DG 34:1	$C_{37}H_{74}O_5N$	16:0_18:1	612.5567	612.5549	-2.9369
DG 36:4	$C_{39}H_{72}O_5N$	18:1_18:3	634.5410	634.5393	-2.7547
DG 36:2	$C_{39}H_{76}O_5N$	18:1/18:1	638.5723	638.5703	-3.2087
DG 34:3;O2	$C_{37}H_{70}O_7N$	16:0_18:3;O2	640.5152	640.5146	-0.9820

Abbreviations: PC: phosphatidylcholine; LPC: lyso phosphatidylcholine; PG: phosphatidylglycerol; PI: phosphatidylinositol; MGDG: monogalactosyl diacylglycerol; MGMG: monogalatosyl monoacylglycerol; DGDG: digalactosyl diacylglycerol; DGMG: digalactosyl monoacylglycerol; SQDG: sulfoquinovosyl diacylglycerol; SQMG: sulfoquinovosyl monoacylglycerol; DGTS: diacylglyceryl-*N*,*N*,*N*-trimethyl homoserine; MGTS: monoacylglyceryl-*N*,*N*,*N*-trimethyl homoserine; Cer: ceramide; PI-Cer: inositolphosphoceramide; TG: triacylglycerols; DG: diacylglycerols.

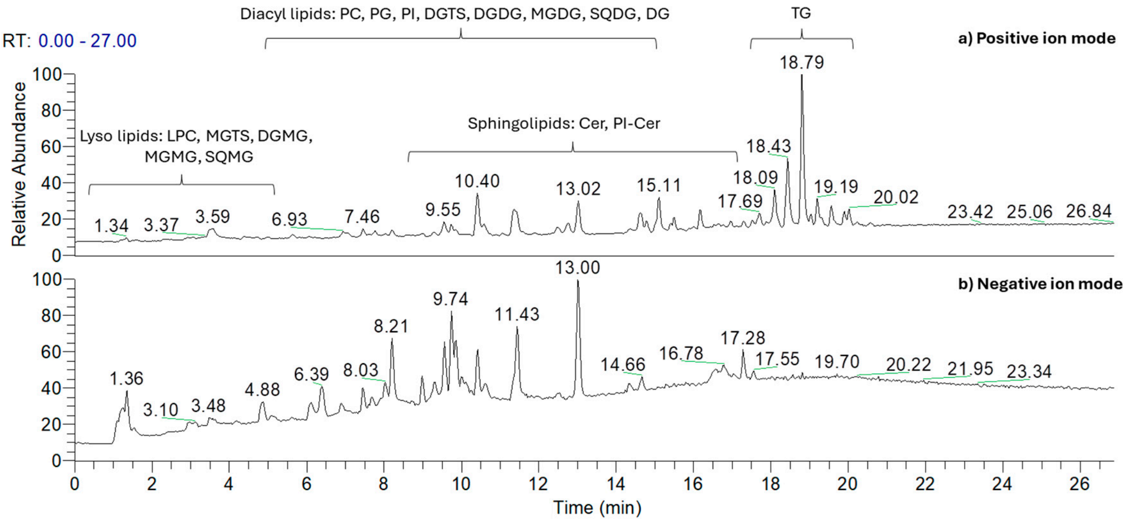

Figure S1: Representative total ion chromatogram (TIC) from C18 reversed-phase liquid chromatography-mass spectrometry (C18 RP-LC-MS) analysis of *Dunaliella salina* lipid extract, shown in positive (a) and negative (b) ion modes. Retention time for the elution of the different lipid classes identified in *D. salina* is also highlighted. Abbreviations: ceramide (Cer), ceramide phosphoinositol (PI-Cer), diacylglyceryl-*N*,*N*,*N*-trimethyl homoserine (DGTS), digalactosyl diacylglycerol (DGDG), digalactosyl monoacylglycerol (DGMG), diglyceride (DG), monoacylglyceryl-*N*,*N*,*N*-trimethyl homoserine (MGTS), monogalactosyl diacylglycerol (MGDG), monogalactosyl monoacylglycerol (MGMG), lyso phosphatidylcholine (LPC), phosphatidylcholine (PC), phosphatidylglycerol (PG), phosphatidylinositol (PI), sulfoquinovosyl diacylglycerol (SQDG), sulfoquinovosyl monoacylglycerol (SQMG), and triglyceride (TG).

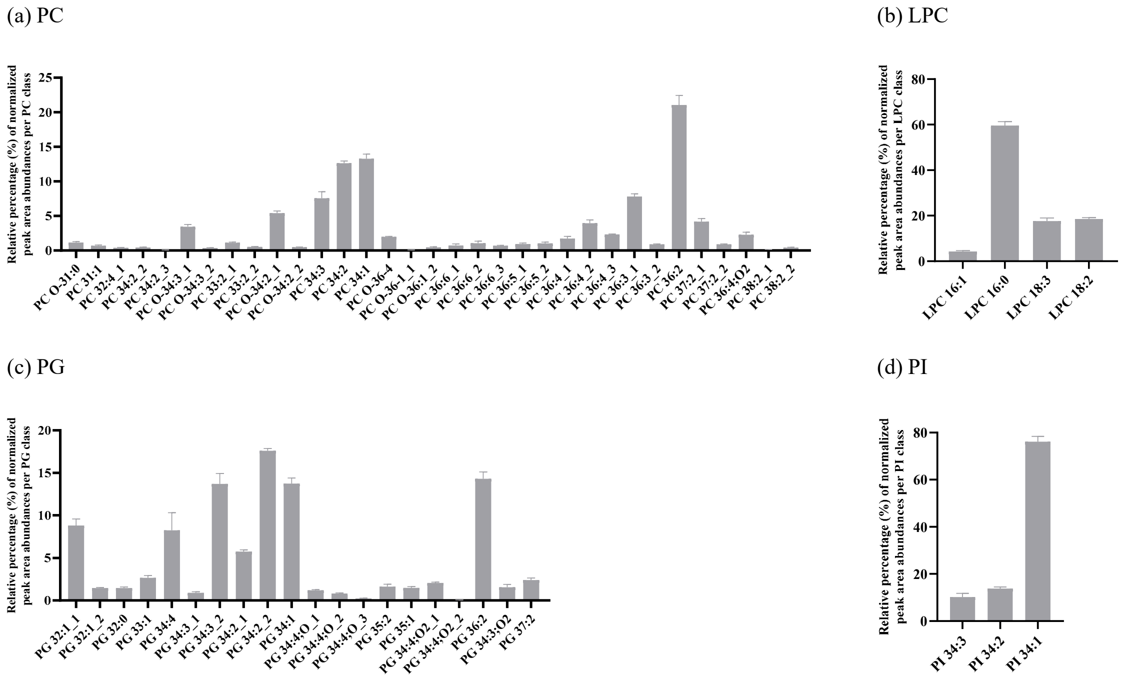

Figure S2: The relative percentage (%) of normalized peak area abundances of identified species per phospholipid class of *Dunaliella salina*. PC (a), LPC (b), PG (c), and PI (d).

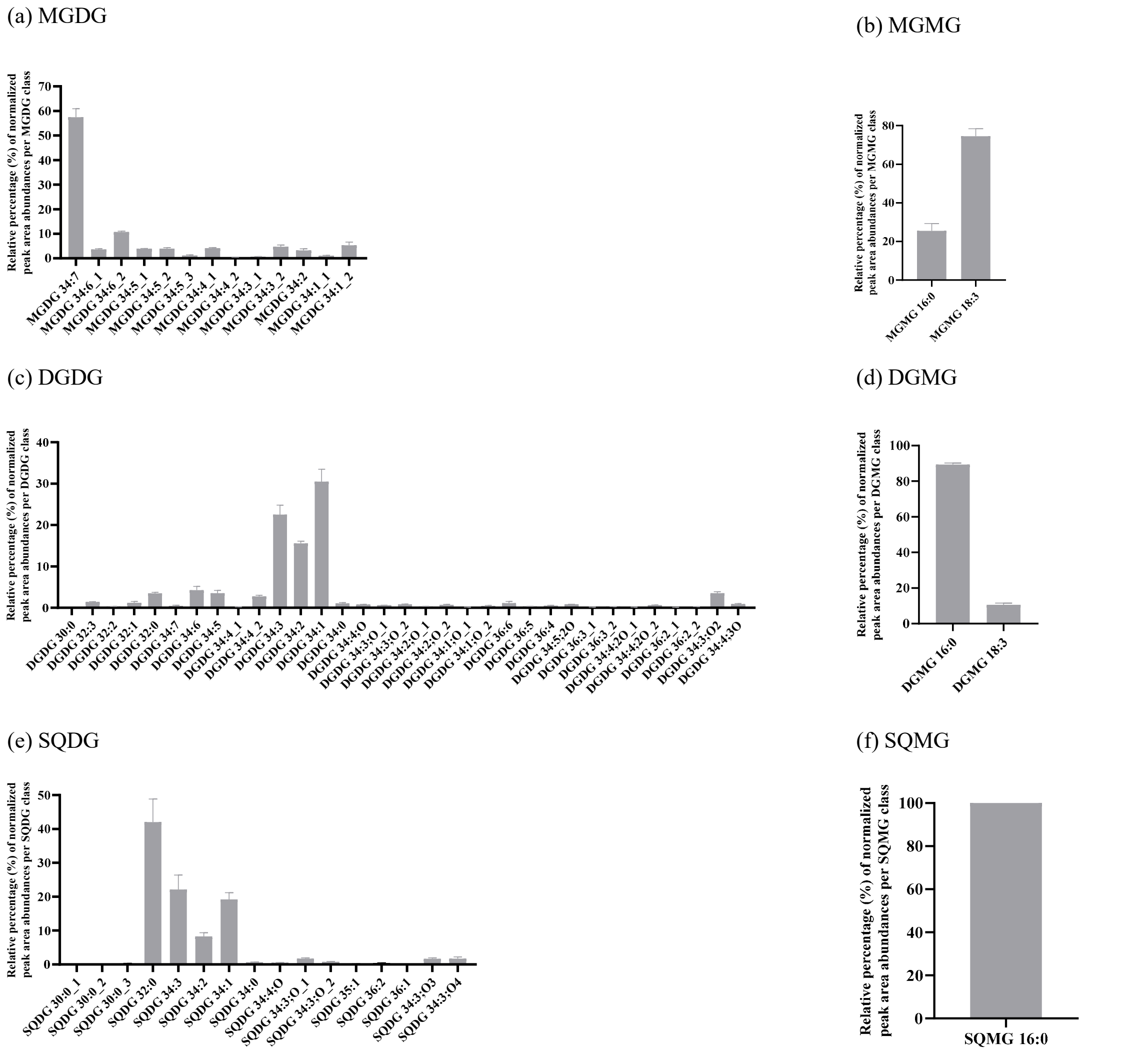

Figure S3: The relative percentage (%) of normalized peak area abundances of identified species per glycolipid class of *Dunaliella salina*. MGDG (a), MGMG (b), DGDG (c), DGMG (d), SQDG (e), and SQMG (f).

(a) DGTS

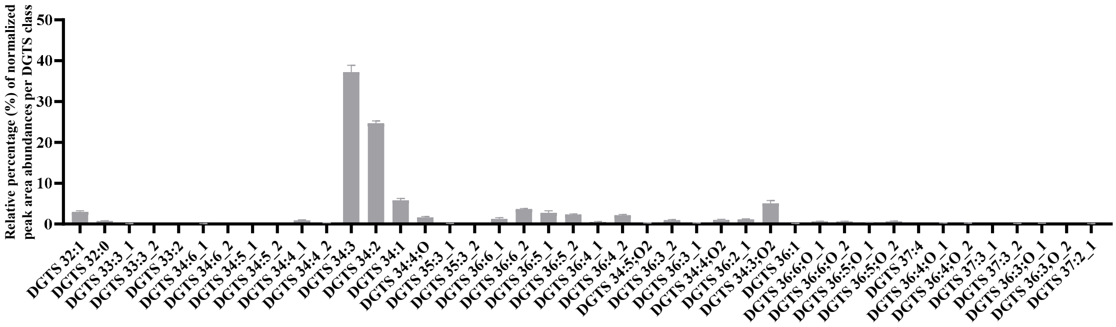

(b) MGTS

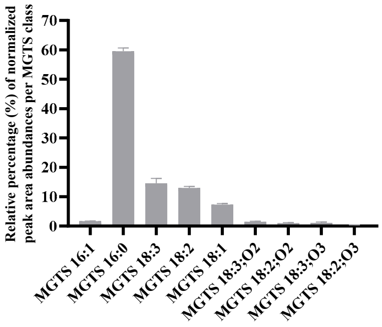

Figure S4: The relative percentage (%) of normalized peak area abundances of identified species per betaine lipid class of *Dunaliella salina*. DGTS (a) and MGTS (b).

(a) Cer

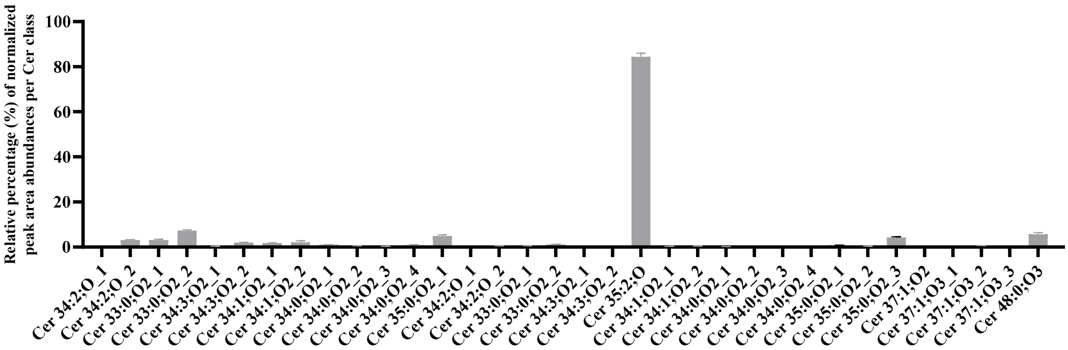

(b) PI-Cer

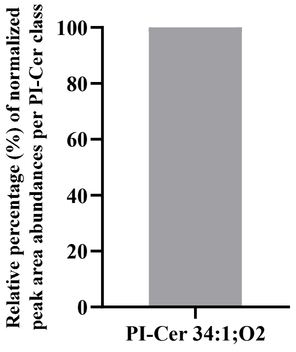

Figure S5: The relative percentage (%) of normalized peak area abundances of identified species per sphingolipid class of *Dunaliella salina*. Cer (a) and PI-Cer (b).

(a) TG

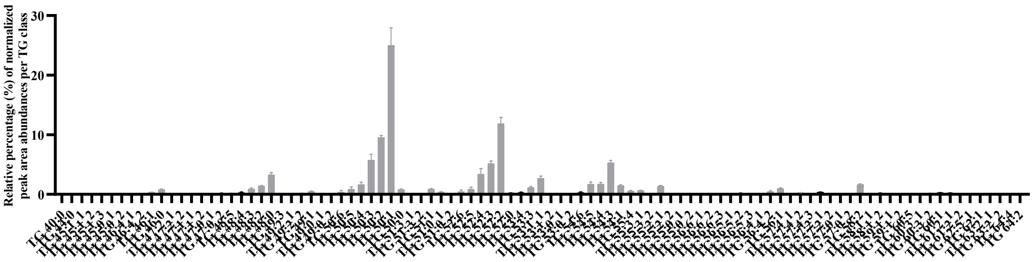

(a1) TG

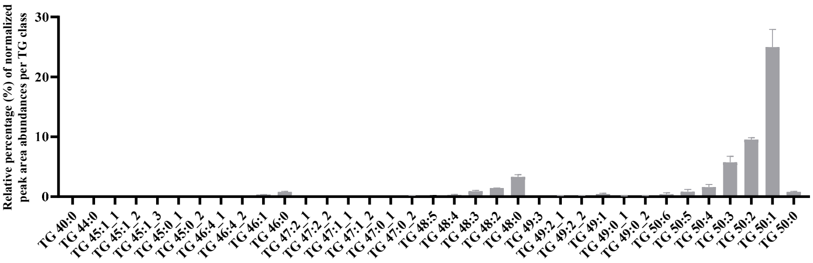

(a2) TG

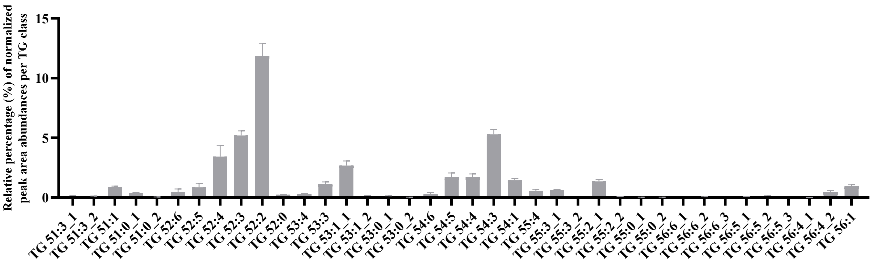

(a3) TG

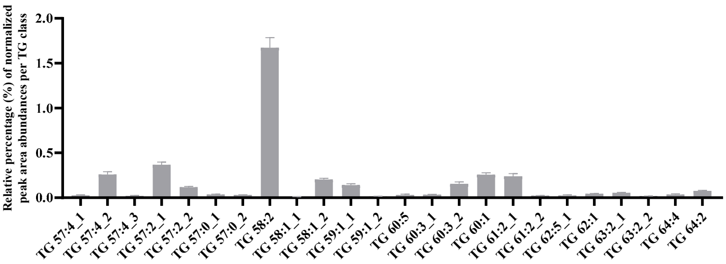

(b) DG

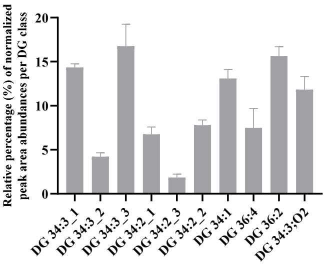

Figure S6: The relative percentage (%) of normalized peak area abundances of identified species per neutral lipid class of *Dunaliella salina*. TG (a) and DG (b).

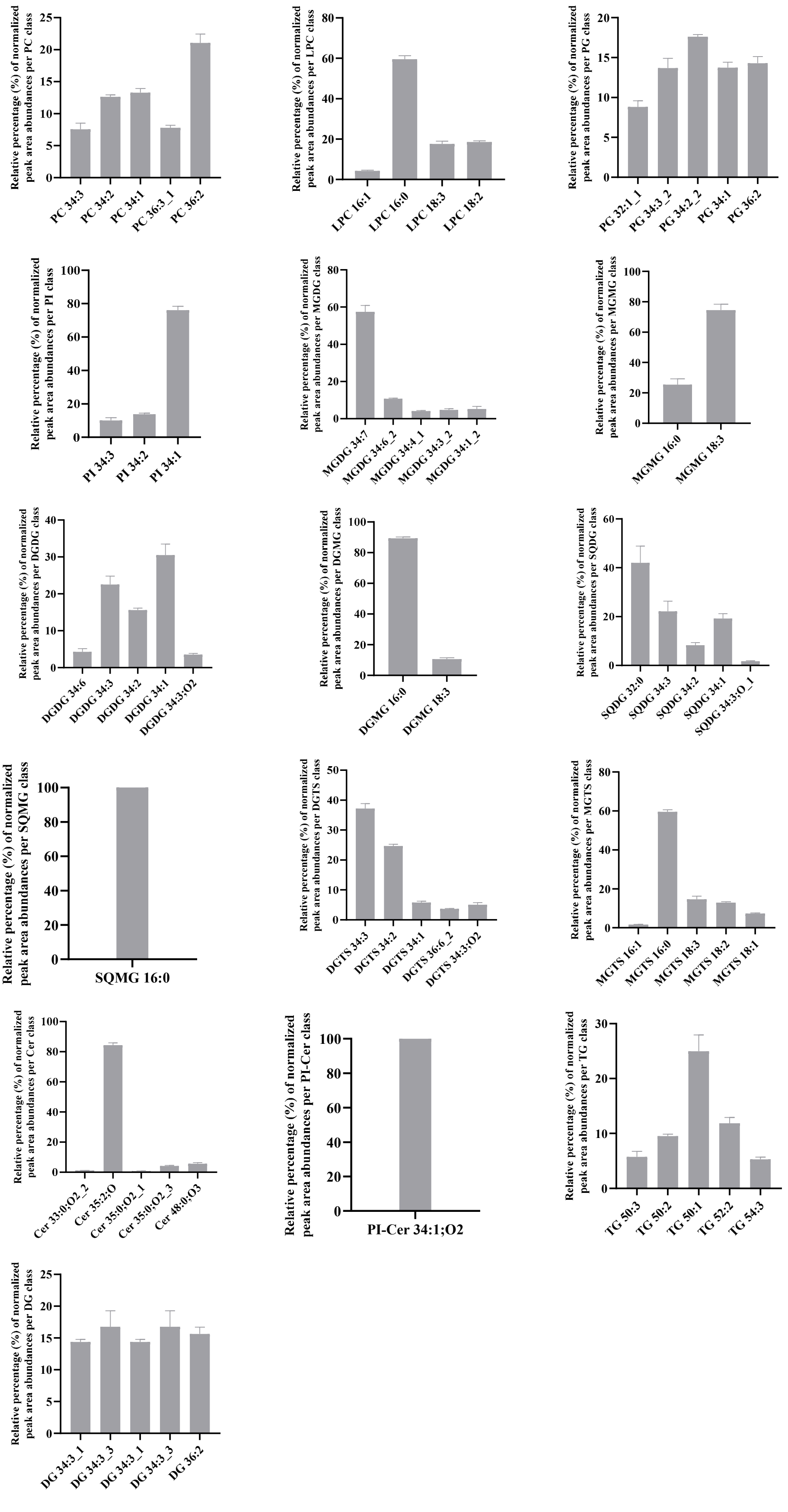

Figure S7: The relative percentage (%) of normalized peak area abundances of the top five most abundant lipid molecular species of each lipid class identified in *Dunaliella salina* lipid extract (LE) using C18 RP-LC-MS.
